# Gene-Metabolite Network Analysis Revealed Tissue-Specific Accumulation of Therapeutic Metabolites in *Mallotus japonicus*

**DOI:** 10.3390/ijms22168835

**Published:** 2021-08-17

**Authors:** Megha Rai, Amit Rai, Tetsuya Mori, Ryo Nakabayashi, Manami Yamamoto, Michimi Nakamura, Hideyuki Suzuki, Kazuki Saito, Mami Yamazaki

**Affiliations:** 1 Graduate School of Pharmaceutical Sciences, Chiba University, Chiba 260-8675, Japan; megha@chiba-u.jp (M.R.); amit.rai@riken.jp (A.R.); manami.y1018@gmail.com (M.Y.); michimi@chiba-u.jp (M.N.); kazuki.saito@riken.jp (K.S.); 2 Plant Molecular Science Center, Chiba University, Chiba 260-8675, Japan; 3 RIKEN Center for Sustainable Resource Science, Yokohama, Kanagawa 230-0045, Japan; tetsuya.mori@riken.jp (T.M.); ryo.nakabayashi@riken.jp (R.N.); 4 Department of Research and Development, Kazusa DNA Research Institute, Kisarazu, Chiba 292-0818, Japan; h_suzuki@hirata.co.jp

**Keywords:** *Mallotus japonicus*, medicinal plants, biosynthesis of specialized metabolites, bergenin, rutin, WCNA, integrative omics, glucosyltransferases, *O*-methyltransferases

## Abstract

*Mallotus japonicus* is a valuable traditional medicinal plant in East Asia for applications as a gastrointestinal drug. However, the molecular components involved in the biosynthesis of bioactive metabolites have not yet been explored, primarily due to a lack of omics resources. In this study, we established metabolome and transcriptome resources for *M. japonicus* to capture the diverse metabolite constituents and active transcripts involved in its biosynthesis and regulation. A combination of untargeted metabolite profiling with data-dependent metabolite fragmentation and metabolite annotation through manual curation and feature-based molecular networking established an overall metabospace of *M. japonicus* represented by 2129 metabolite features. *M. japonicus* de novo transcriptome assembly showed 96.9% transcriptome completeness, representing 226,250 active transcripts across seven tissues. We identified specialized metabolites biosynthesis in a tissue-specific manner, with a strong correlation between transcripts expression and metabolite accumulations in *M. japonicus*. The correlation- and network-based integration of metabolome and transcriptome datasets identified candidate genes involved in the biosynthesis of key specialized metabolites of *M. japonicus*. We further used phylogenetic analysis to identify 13 C-glycosyltransferases and 11 methyltransferases coding candidate genes involved in the biosynthesis of medicinally important bergenin. This study provides comprehensive, high-quality multi-omics resources to further investigate biological properties of specialized metabolites biosynthesis in *M. japonicus*.

## 1. Introduction

*Mallotus japonicus*, also known as “Akamegashiwa” in Japanese, is a well-known medicinal plant from the Euphorbiaceae family and is widely distributed in tropical and temperate parts of East Asia [1,2,3,4]. Different tissues of *M. japonicus*, such as leaves, root, bark, and pericarp, have been shown to be effective in several ailments, including gastric and duodenal ulcer, hyperacidity, irritable bowel syndrome, hemorrhoids, rheumatism, diabetes, dermatitis, liver diseases, and neuralgia [1]. The bark of *M. japonicus* is still listed as a stomach tonic for improving appetite in the “17th revision of the Japanese Pharmacopoeia”. Bark tissue has been traditionally used to treat stomach disorders and gallstones, as a folk medicine for cancer, and as a crude drug for gastric and duodenal ulcers [5].

The medicinal properties of a given plant are attributed to its metabolic constituents, which include bioactive metabolites, and are often accumulated in a tissue-specific manner. Currently, over 143 compounds have been isolated across different *Mallotus* species, including 99 compounds in *M. japonicus*. These compounds belong to different chemical families such as alkaloids, iridoids, flavonoids, polyphenols, cardenolides, phloroglucinol, tannins, and their derivatives [1,2]. The leaves of *M. japonicus*, which primarily contains rutin and isoprenoid derivatives, are used to treat boils and swellings [5]. Furthermore, the antioxidative properties of *M. japonicus* leaves and their components have been evaluated, and this plant is used as a functional food for human health. Bergenin, a dihydroisocoumarin derivative, possesses anti-inflammatory, antitussive, and hypolipidemic activity; is known for its effectiveness in treating gastritis, gastric ulcer, constipation, and diarrhea [6,7,8,9,10]; and is specifically accumulated in the bark of *M. japonicus* [11]. Pharmaceuticals such as gastrointestinal drugs have included *M. japonicus* as a key ingredient for over 45 years. Despite vast industrial and medicinal applications, including a potential source of new medicines, no comprehensive multi-omics studies have ever been reported for *M. japonicus*, thus resulting in the lack of genomics and metabolomics resources for this plant species [1,2].

The advancement of high-throughput-omics technologies has facilitated the generation of a wealth of knowledge. The omics datasets for a given species are valuable. When analyzed individually, associations with other omics datasets and comparisons across species provide a holistic view of a biological species [12,13]. Untargeted metabolomics is a “discovery mode” process that gives insight into each metabolite, whether known or unknown, present in the biological entity under study [14]. Additionally, with the rapid development of sequencing technologies, RNA-seq based de novo transcriptome assembly has been established as a powerful tool to study non-model plant species with little or no genomic resources [12,13]. Transcriptome profiling provides an extensive overview of the metabolic pathways and their localization in planta. At the same time, the sequence-similarity approach, together with distinct statistical analyses, assists in the identification of candidate genes involved in the biosynthesis of important metabolites. When combined with phylogenomic, such approaches allow for the identification of potential enzymes involved in the biosynthesis of specialized metabolites.

**Figure 1:**
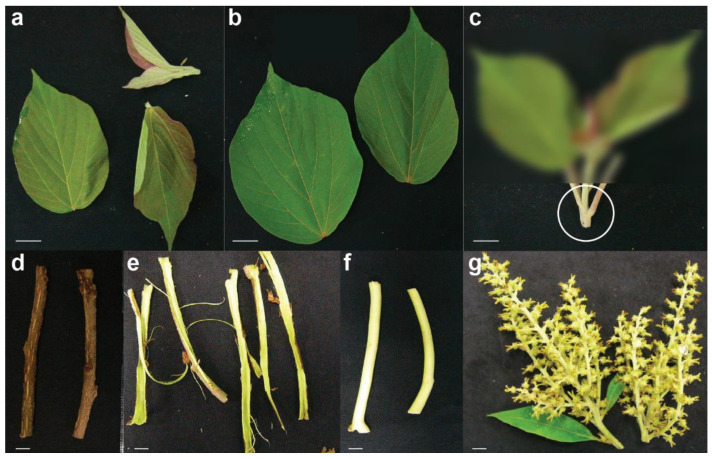
Tissues of *Mallotus japonicus* used in this study for the multi-omics analysis: (**a**) young leaf, (**b**) mature leaf, (**c**) young stem, (**d**) mature stem, (**e**) bark, (**f**) central cylinder, and (**g**) inflorescence. The scale bar represents 1 cm.

To establish a comprehensive metabolite repository and genomic resources for *M. japonicus*, we performed untargeted metabolite profiling and deep-sequencing using seven tissues, namely young leaf, mature leaf, young stem, mature stem, bark, central cylinder, and inflorescence (Figure 1). Untargeted metabolomics helped us capture the differential accumulation pattern of several specialized metabolite classes present in *M. japonicus*. WCNA-based network analyses were performed using the expression data of assembled transcripts and intensities of MS^2^ validated metabolites to classify metabolites or genes with similar functionalities into distinct modules. Furthermore, a pairwise correlation between the transcript and metabolite modules was performed to identify gene-to-metabolite associations highlighting the potential candidate genes involved in the biosynthesis of specialized metabolites. Our study identified a distinct transcript module involved in the biosynthesis of rutin in *M. japonicus*. Furthermore, we performed a gene ontology enrichment analysis to understand the biological significance of the transcript modules having a high correlation with the metabolic modules, which showed the presence of functional modules specific for the type of metabolites accumulated. The metabolome and transcriptome data established in this study will help in understanding the biosynthesis and regulation of specialized metabolites in *M. japonicus*.

## 2. Results and Discussion

### 2.1. Mallotus japonicus Metabolome Database Represents Diverse Range of Specialized Metabolites

In order to achieve holistic insight into the phytochemical composition of *M. japonicus*, the metabolite extracts from seven tissues, namely young leaf, mature leaf, young stem, mature stem, bark, central cylinder, and inflorescence (Figure 1), were analyzed by LC–QTOF–MS. Furthermore, feature-based molecular networking (FBMN) was employed to characterize the metabolite classes captured by our comprehensive metabolome data. This approach has recently gained attention in the annotation of the chemical diversity captured by untargeted metabolite profiling [15,16]. Molecular networking not only matches the MS^2^ spectra for a given mass feature to assign a putative annotation but also connects molecules based on spectral similarity to predict and assign mass features to its derivatives or chemical classes, which differs by transformation including glycosylation, hydroxylation, oxidation, alkylation, and reduction, among others [15,16].

**Figure 2:**
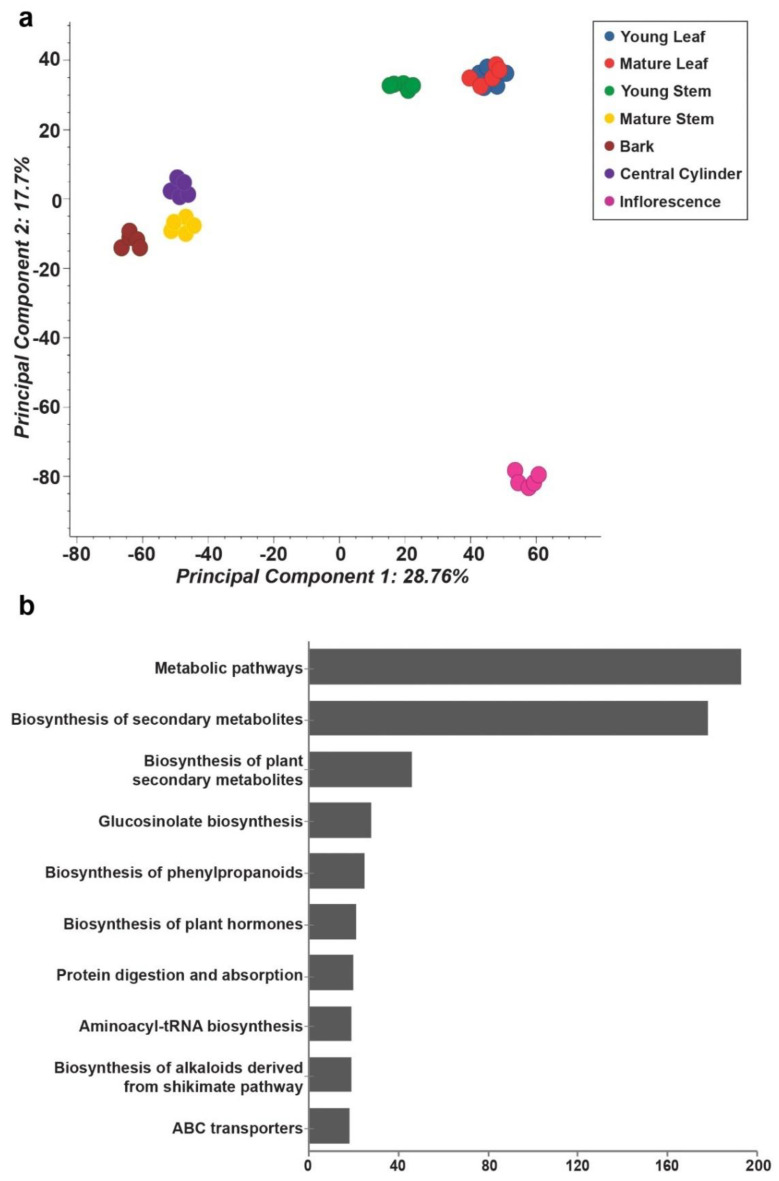
Untargeted metabolome analysis for seven tissues of *Mallotus japonicus*. (**a**) Principal component analysis using the metabolome datasets acquired for seven tissues of *M. japonicus*; (**b**) The top 10 KEGG pathways based on the number of metabolites assigned. The chemical identities of the mass features and corresponding KEGG compound ID were assigned based on a narrow mass-error window of 10 ppm, and candidate metabolites were grouped according to the assigned KEGG pathway category.

The metabolite profile datasets from seven tissues of *M. japonicus* were pre-processed and aligned using MS-DIAL [17], resulting in the identification of 10,367 mass features in the positive ion mode (Table S1). Principal component analysis (PCA) using filtered and aligned mass features showed tissue-specific variations and formed three major groups (Figure 2a). Young leaf, mature leaf, and young stem formed a group separated along the PC1 axis, with mature stem, bark, and central cylinder grouped together. Inflorescence tissue formed a distinct group and was separated from other tissues along the PC2 axis. A PCA plot confirmed the quality of metabolome datasets that effectively captured the tissue-specific metabolite diversity. In an attempt to understand the overall phytochemical constituents and the active metabolic pathways of *M. japonicus*, the acquired mass-features were searched against the KNApSAcK database [18] and KEGG pathway database [19], respectively. In total, 2129 and 564 mass features were assigned to a corresponding KNApSAcK ID and KEGG ID, respectively (Table S2). The top three KEGG pathways based on the number of mass features assigned included metabolic pathways, biosynthesis of secondary metabolites, and biosynthesis of plant secondary metabolites (Figure 2b). Several secondary metabolic pathways, including glucosinolate biosynthesis, biosynthesis of phenylpropanoids, biosynthesis of alkaloids derived from shikimate pathways, and flavonoid biosynthesis were included in the top 15 KEGG pathways. These results indicate a plethora of diverse metabolites in *M. japonicus*, of which only 143 are reported to date [2]. The *M. japonicus* metabolome database thus represents a repository of novel, unknown metabolites that could explain its specific medicinal properties as employed in traditional medical practices.

**Figure 3:**
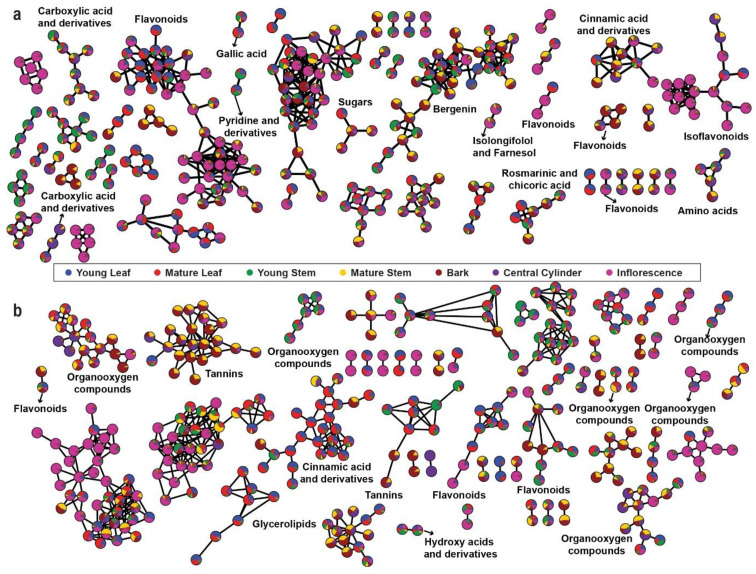
Feature-based Molecular Networking (FBMN) using the metabolome datasets acquired in the (**a**) positive mode and (**b**) negative mode. The pie charts on the nodes are drawn according to the accumulation pattern of the assigned metabolites across seven tissues of *Mallotus japonicus*.

The aligned mass features with MS^2^ fragmentation pattern were used for FBMN analysis using GNPS platform (http://gnps.ucsd.edu//, accessed on 30 November 2020). Two molecular networks were generated using the MS^2^ spectra of parent ions in the positive and negative ionization modes. The nodes in the network are represented as a pie chart to reflect the intensity level across seven tissues of *M. japonicus* (Figure 3a,b). For the positive ion mode, 305 connected nodes were identified (with a minimum of two connected components), and 80 nodes were annotated to represent different chemical families (Table S3). The highly connected nodes included mass features identified as flavonoids, isoflavonoids, carboxylic acid and derivatives, and cinnamic acid and derivatives (Figure 3a). The results were expected since these metabolite classes are well known to undergo chemical modifications and derivatizations, resulting in vast chemical diversity from relatively fewer core metabolites [20,21,22]. The bergenin-containing cluster was represented by 35 metabolite features, indicating closely related metabolite features of bergenin in *M. japonicus*. While the node containing flavonoids and isoflavonoids was highly accumulated in inflorescence, the node containing bergenin and related metabolites was highly accumulated in bark. The molecular network generated using the negative ionization mode resulted in 330 connected components, and 68 nodes were annotated (Table S3). While two highly connected nodes were not annotated, the third significantly connected node included metabolite features classified as tannins and was highly accumulated in the bark. The highly connected nodes, with mass features identified as cinnamic acid and derivatives, were highly accumulated in the mature leaf of *M. japonicus* (Figure 3b). Untargeted metabolite profiling using seven tissues of *M. japonicus* followed by characterization and annotation of the acquired mass-features revealed the presence of a diverse range of phytochemicals attributed to its broad range of medicinal uses. Moreover, the molecular network based on the fragmentation pattern also showed a large number of highly connected nodes that remained unannotated, which indicates the presence of several metabolites not reported in *M. japonicus*. Therefore, the comprehensive metabolome resource established in this study is a valuable resource that can be further used to unfold the pharmacological potentials of several unreported metabolites and to serve as a stepping-stone for future investigation and structural validation of the bioactive metabolites in *M. japonicus*.

### 2.2. Tissues of Mallotus japonicus Have Specific Metabolic Signatures Associated with Its Medicinal Use

**Figure 4:**
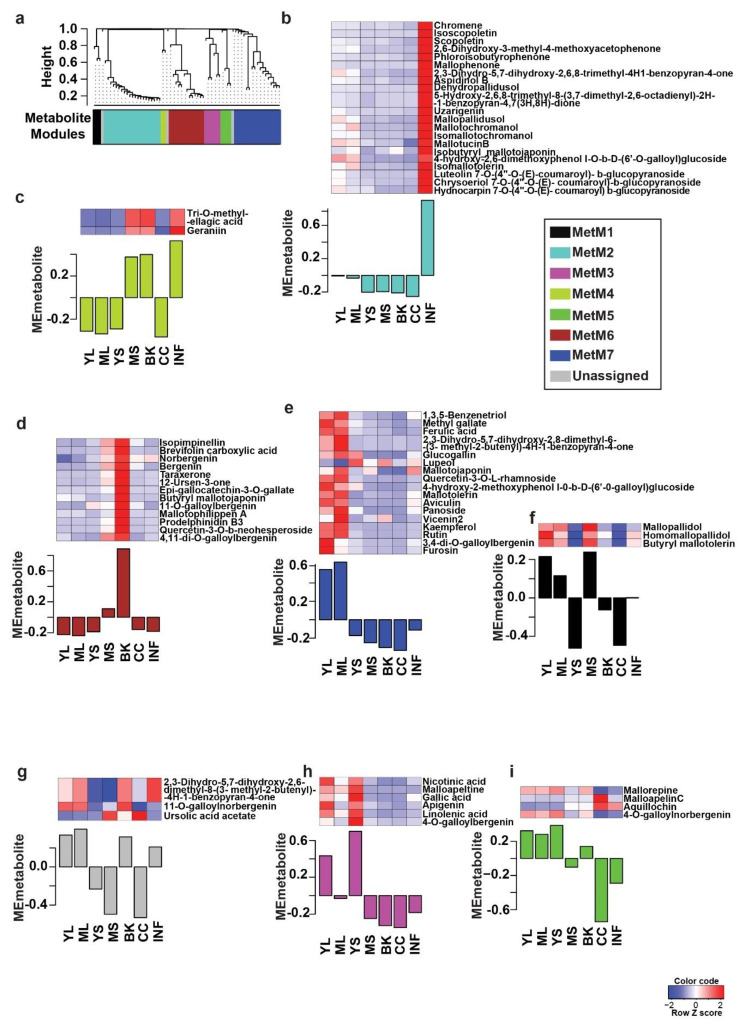
WCNA analysis and accumulation pattern of MS^2^ confirmed the metabolites in *Mallotus japonicus*. (**a**) The dendrogram represents 69 MS^2^-confirmed metabolites used for WCNA analysis and clustering of module eigenmetabolites; (**b**–**i**) The bar plots show the module eigenmetabolite of MS^2^-confirmed metabolites. The relative accumulation levels of metabolites included in each module are also shown by the heat maps above each bar plot. Abbreviations: ML: Mature leaf, YS: Young stem, YL: Young leaf, CC: Central cylinder, MS: Mature stem, INF: Inflorescence, and BK: Bark.

The comprehensive metabolome resource established for *M. japonicus* in this study was subsequently used to identify and validate specialized metabolites reported for the *Mallotus* genus to date. Through an extensive literature search, we short-listed 143 metabolites previously reported across the *Mallotus* genus. Using a combination of the computational cheminformatics approach, manual inspection, and validation, we confirmed 69 metabolites across different tissues of *M. japonicus* by matching their MS^2^-based fragmentation pattern (Table S4). The variations within the MS^2^ validated metabolites indicated the tissue-specific accumulation; therefore, we applied a weighted co-expression network analysis (WCNA) using these 69 metabolites [23,24]. WCNA, a correlation-based method, defines a network linking high-dimensional datasets, including metabolomics data, gene expression data, and proteomics data, and further groups the highest co-expressed variables into modules [23,25,26]. The WCNA approach classified 66 metabolites in seven metabolite modules (MetM), while three metabolites were not assigned to any of the modules (shown with gray bars) (Figure 4). The MetM2, MetM7, and MetM6 modules were the three largest metabolite modules, representing 21, 17, and 13 metabolites, respectively (Table S5). The MetM2 module, which primarily included phloroglucinols (47.6%), was highly accumulated in the inflorescence tissue. Metabolites in the MetM7 module, which included flavonoids such as the rutin, tannin, and phloroglucinol classes of metabolites, were primarily accumulated in the young and mature leaf. The MetM6 module, which included bergenin and its derivatives, one of the most important pharmacological metabolites for *Mallotus* species, was highly accumulated in bark tissue in accordance with the primary tissue used for medicinal purposes. The MetM3 module containing six metabolites, representing various metabolite classes, was primarily accumulated in young stem followed by young leaf. The MetM5 module included four metabolites, two of which were annotated as phenylpropanoids, that were highly accumulated in young stem followed by young and mature leaf. The MetM1 module, which included three of the metabolites of phloroglucinol class, was highly accumulated in mature stem followed by young leaf and mature leaf. The MetM4 module, which included only two tannins, was highly accumulated in inflorescence, bark, and mature stem. Rutin, which is the major active constituent of the leaf tissue of *M. japonicus* and the attribute contributing to its medicinal properties, was found to be highly accumulated in both young leaf and mature leaf in our study [27,28]. Similarly, bergenin and its derivatives were highly accumulated in the bark, as reported previously [28]. The WCNA analysis of the MS^2^ validated metabolites thus effectively captured and classified the signature metabolites associated with the tissue types of *M. japonicus*. The wide range of metabolites accumulated across different tissues of *M. japonicus* reiterates their great medicinal potential, which still seems relatively unexploited, as suggested by the large number of unidentified mass features reported in our study.

### 2.3. Characteristics of Mallotus japonicus De Novo Transcriptome Assembly Derived Using Multiple Tissues

The availability of genomic resources is the prerequisite to evaluate molecular components involved in specialized metabolites biosynthesis. While the number of plant species with chromosome-scale genome assemblies has significantly increased in the last few years, the sequenced genomes are still dominated by crops, and relatively fewer medicinal plants have obtained the high-quality genome status so far [13,29]. The scarcity of genomic resources for medicinal plants has hindered the prospect of developing a sustainable alternative to produce pharmacological metabolites of plant origin. The recently reported 1 KP dataset showed the efficacy of RNA-seq based genome resources in exploring the relationships between distant species [30]. However, it has not even captured a fraction of the known plant diversity. RNA-seq based transcriptome assembly does not require the availability of reference genome, and its application has been well established for studying the biosynthesis of specialized metabolites in medicinal plants [30,31,32,33,34]. In this study, to capture the active transcripts involved in the biosynthesis of specialized metabolites, we established a RNA-seq based de novo transcriptome assembly for *M. japonicus* using the same seven tissues used for the metabolome analysis. The sequencing reads were preprocessed using Trimmomatic [35] (Table S6) and were assembled using a combination of primary assemblies generated through Trinity [36], SOAPdenovo-Trans [37], and CLC genomics workbench, as described previously [38]. From a total of 15.59 Gbp sequencing datasets across seven tissues, the final assembly resulted in 226,250 unigenes with an N50-value of 1396 bp (Table 1). We evaluated the quality and completeness of the de novo transcriptome assembly of *M. japonicus* using a BUSCO analysis [39]. The BUSCO analysis using the eudicots_odb10 database estimated 96.9% assembly completeness, with 2255 out of 2326 BUSCO groups being identified in the *M. japonicus* transcriptome assembly (Figure S1a). Within the identified BUSCO orthogroups, 828 (35.5%) were complete and single-copy BUSCOs, 1427 (61.3%) were completed and duplicated BUSCOs, and only 39 (1.4%) were fragmented BUSCOs. As the final de novo transcriptome assembly was processed with CD-HIT-EST [40]-based redundancy filtering, a high percentage of duplicated BUSCO groups may suggest a potential whole-genome duplication event for *M. japonicus*. The BUSCO analysis results suggest a high-quality transcriptome assembly of *M. japonicus* established in this study, suitable for further downstream applications. The length of the assembled transcripts ranged from 200 bp to 17,202 bp, with a mean length of 837 bp (Figure S1b).

The assembled unigenes of *M. japonicus* were used as a query to perform a Blastx-based homology search against the NCBI non-redundant (nr) database, and the top blast-hit was selected for annotation. Of the total 226,250 unigenes, 109,178 were annotated based on their Blastx-based similarity against the NCBI database (Figure S2a and Table S7). The similarity distribution plot for the annotated unigenes showed 64,021 unigenes (~25% of entire unigenes) with a sequence-similarity over 85% with its closest homolog (Figure S2b). The Gene Ontology (GO)-based functional classification assigned annotated transcripts to three GO categories, namely biological process (BP), molecular function (MF), and cellular component (CC). The top 10 GO terms based on the number of mapped sequences are summarized in Figure S2c. Furthermore, we mapped the annotated unigenes to the KEGG pathway database [19] to derive an overview of the active metabolic processes of *M. japonicus*. In total, 17,434 unigenes were mapped to 148 KEGG pathways (Table S8). The top 15 KEGG pathways based on the number of mapped unigenes are shown in Figure S2d. The top 5 pathways included glycolysis/gluconeogenesis, starch and sucrose metabolism, purine metabolism, glyoxylate and dicarboxylate metabolism, and pyruvate metabolism. The phenylpropanoid biosynthesis was among the top 10 metabolic pathways represented in the transcriptome assembly of *M. japonicus*. The phenylpropanoid class of metabolites have been widely reported in *M. japonicus* and were also identified as one of the dominant class of metabolites in our metabolomics dataset. The annotation and characterization of *M. japonicus* de novo transcriptome assembly effectively captured ongoing active metabolic processes in accordance with the class of metabolites being identified in our metabolome analyses. Therefore, our results demonstrate the suitability of our transcriptome resources established in this study for downstream analyses.

We used de novo transcripts assembly to qualitatively measure the expression levels of active transcripts across seven tissues of *M. japonicus*. RNA-sequencing reads from each of the seven tissues were mapped onto the transcriptome assembly, and the expression of assembled transcripts was measured in terms of FPKM values. Among the seven tissues, bark and inflorescence, with 158,610 (70.1%) and 154,492 (68.9%) transcripts, respectively, represented the highest number of transcriptionally active unigenes. In contrast, mature leaf and young stem, with 144,210 (63.7%) and 145,483 (64.3%) transcripts, respectively, showed the lowest number of transcriptionally active unigenes (Figure S3a and Table S9). Bark and inflorescence tissue, having the highest number of transcriptionally active unigenes, also showed a high accumulation pattern for several of the MS^2^-validated metabolites identified in our study (Figure 4). Unsupervised principal component analysis using transcripts’ expression grouped the seven tissues into four major groups (Figure S3b). Bark and mature stem were grouped together and separated along the PC1 axis with the second group including young leaf, mature leaf, and young stem. The inflorescence and central cylinder formed separate clusters. The transcriptome expression analysis together with PCA analysis suggests that our transcriptome data effectively captured the ongoing specific as well as common metabolic processes across each tissue of *M. japonicus* and hence can be used as a valuable resource to further analyze and identify candidate genes participating in the biosynthesis of specialized metabolites in *M. japonicus*.

### 2.4. Network-Based Characterization of Transcriptome and Metabolome Relationships in Mallotus japonicus

**Figure 5:**
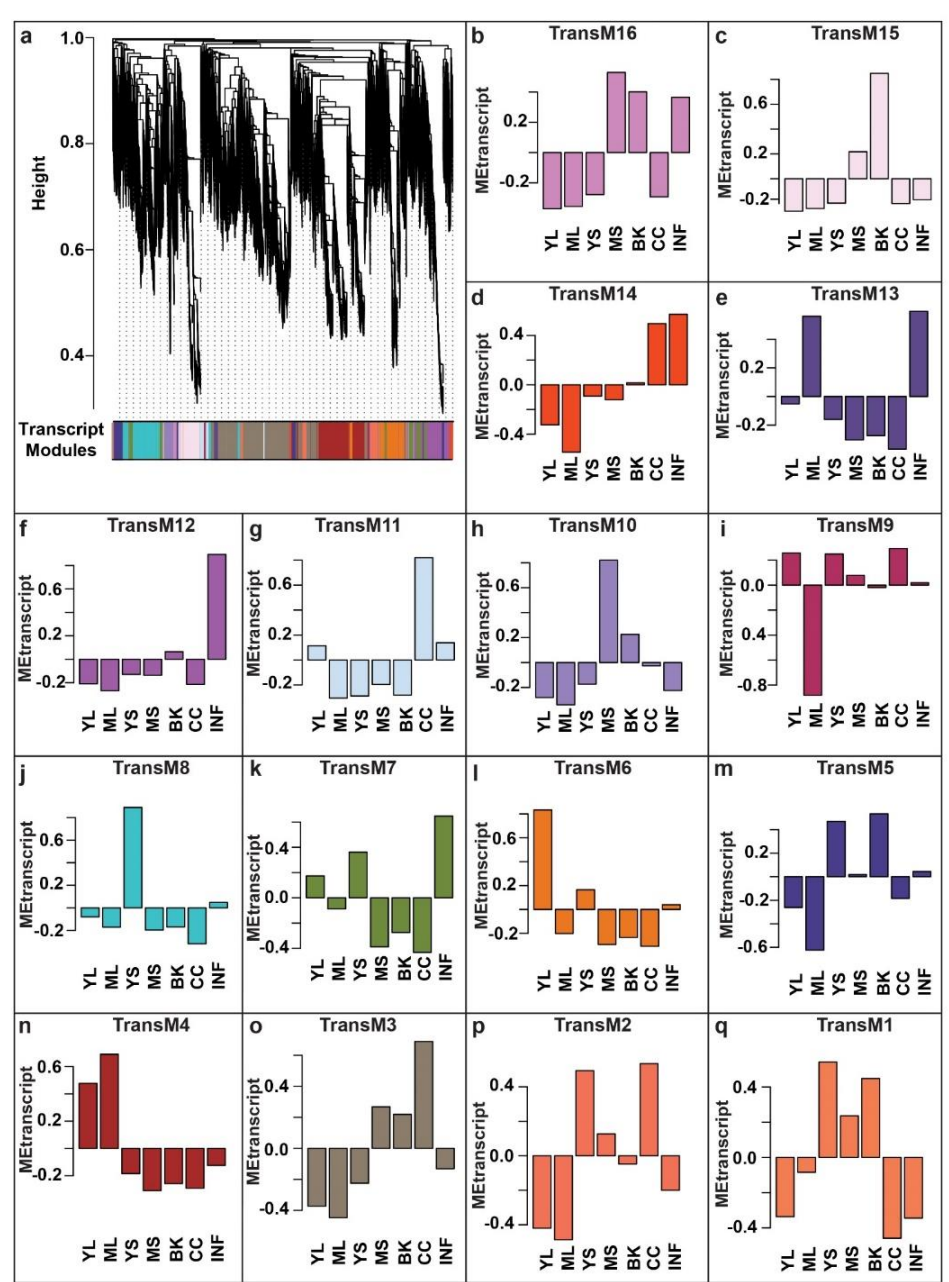
WCNA-based expression analysis using highly expressed transcripts in at least one of the seven tissues of *Mallotus japonicus*. (**a**) A dendrogram of the transcripts used for WCNA analysis and clustering of module eigentranscripts; (**b**–**q**) The bar plots show the eigenvalue of the transcript modules, which summarizes the first principal component of the given trans module. Abbreviations: ML: Mature leaf, YS: Young stem, YL: Young leaf, CC: Central cylinder, MS: Mature stem, INF: Inflorescence, and BK: Bark.

Gene co-expression networks derived through WCNA analysis have emerged as a robust systems biology approach to discover associations within biomolecules [41,42,43,44,45,46,47,48]. As demonstrated by the guilt-of-association concept, genes with similar expression patterns are most likely to be associated with a similar biological process [49,50]. Using WCNA, we classified high-dimensional transcriptome datasets into a framework of co-expression modules and explored those transcript modules that may be related to the metabolites’ variation across different tissues. To understand the molecular mechanisms involved in the biosynthesis of specialized metabolites, we used highly expressed and annotated transcripts of *M. japonicus* to perform WCNA analysis (Table S10). In total, we identified 16 transcript modules (TransM), representing clusters of correlated transcripts (Figure 5, Table S11). TransM3 module represents the largest module with 7988 correlated transcripts, while the smallest module, TransM1, included only 147 transcripts, with the mean and median of the assigned transcripts to the transcript modules as 1906 and 1482, respectively. Similar to the tissue-specific accumulation pattern observed in the metabolite modules, the identified transcript modules also showed a tissue-specific expression pattern of the associated transcripts. Among the 16 identified transcript modules, TransM4, TransM6, TransM8, TransM10, TransM12, and TransM15 were specific to mature leaf, young leaf, young stem, mature stem, inflorescence, and bark, respectively, while transcript module TransM3 and TransM11 were specific to the central cylinder tissue (Figure S4).

**Figure 6:**
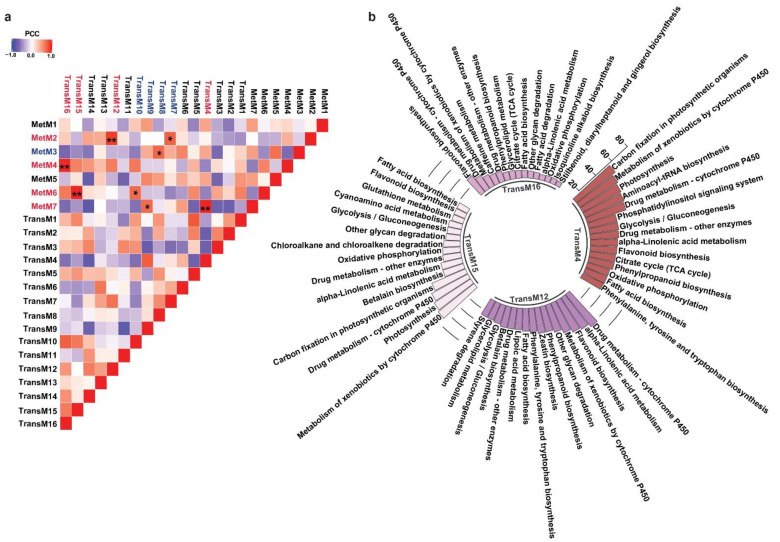
Integrated analysis of transcript and metabolite modules. (**a**) Pearson correlation coefficient between each pair of TransM and MetM module. The red font represents transcript-metabolite modules with over 0.9 correlation, while the blue font represents transcript-metabolite modules with over 0.7 correlation. ** pairs with PCC > 0.9, * PCC > 0.7; (**b**) Top 15 KEGG pathways based on the percentage of specific pathways represented by transcripts co-clustered in TransM4, TransM12, TransM15, and TransM16, the four modules with high correlation with metabolite module.

One of the major conclusions derived from the multi-omics analysis across diverse plant species is the strong correlation between metabolites accumulation and the expression levels of enzymes associated with its biosynthesis and regulation [51]. The correlation-based associations have been particularly prevalent for secondary metabolism and have served as the basis for the functional characterization of thousands of genes to date [52]. While homology-based transcripts annotation often leads to the assignment of same enzyme function to multiple transcripts, primarily due to the prevalent genomic redundancies in plants, not all transcripts are functional [12]. A co-expression analysis does group genes within a cluster, the number of genes within its assigned group is still higher for screening candidate genes for further functional characterization. The metabolic network-driven approach by integrating metabolome and gene co-expression datasets has contributed exceptionally to identifying genes and their associations with a given metabolite. It further narrows down candidate genes for functional characterization and to study the biosynthetic machinery of specialized metabolites in medicinal plants [53]. To understand the biosynthetic network of specialized metabolites in *M. japonicus*, we performed a Pearson correlation analysis between the identified metabolites and transcripts modules using the average value of expression or accumulation of transcripts or metabolites assigned to a given module, respectively, as described previously [54] (Table S12). The highest correlation was obtained for four transcript-metabolite module pairs, namely TransM15–MetM6, TransM4–MetM7, TransM16–MetM4, and TransM12–MetM2, with correlation values of 0.986, 0.986, 0.98, and 0.916, respectively (Figure 6a). To understand their functional classification, we performed KEGG pathway mapping for the transcripts included in each of the four highly correlated transcript modules. The top 15 KEGG pathways based on the percentage of the specific pathway represented by the unigenes are shown in Figure 6b. In total, six of the top 15 pathways were shared between all four modules. The flavonoid biosynthesis pathway was present in all four transcript modules, but the percentage of the total genes of the flavonoid pathway being represented by transcripts from these four modules were significantly different, ranging from as low as 18.4% in the TransM15 module to 54.54% in the TransM12 module (Table S13). On the other hand, phenylpropanoid biosynthesis was represented by transcripts from three modules, including TransM4 (40%), TransM12 (40%), and TransM16 (13.3%) transcript modules (Figure 6b and Table S13). Over 80% of the MS^2^-validated phenylpropanoid and flavonoid classes of metabolites were present in these four metabolite clusters, thus suggesting that the transcripts included in the four correlated transcript modules may represent important classes of enzymes involved in the biosynthesis of these specialized classes of metabolites. To associate GO-based functional classification to the transcript modules, we constructed an interrelated network of the transcript modules highly correlated with the metabolite modules with their top three enriched GO terms from each of the three categories (Figure S5). For the TransM4 module, one of the top 10 GO terms that was enriched included the glucosinolate biosynthetic process, which plays a major role in plant resistance to biotic stress (Table S14). Several of the GO terms related to secondary metabolites, including anthocyanin-containing compound biosynthetic process, lignin catabolic process, and coumarin biosynthetic process, were significantly enriched in the TransM4 module. The transcript module TransM4 was highly correlated with the MetM7 metabolite module, which included several secondary metabolite classes, including flavonoids, tannins, and phloroglucinols. The TransM16 module, which was highly correlated with metabolite module MetM4, showed an enrichment of GO terms including oxidation–reduction process, suberin biosynthetic process, response to nitrate, and nitrate transport. The enriched GO terms for the TransM15 module included oxidation–reduction processes, ethanol catabolic processes, threonine catabolic processes, and responses to sucrose, among others. MetM6, which was highly correlated with TransM15, included bergenin among several other metabolites with high accumulation levels in bark tissue. GO terms related to growth and development, including microtubule cytoskeleton, cotyledon vascular tissue pattern formation, and multidimensional cell growth, were also enriched in the TransM15 module. The TransM12 module showed enrichment of the GO terms involved in plant defense, such as response to chitin, salicylic acid biosynthetic processes, and response to wounding. We also found several GO terms being enriched and related to the biosynthesis of secondary metabolites, including the coumarin biosynthetic process, flavonol biosynthetic process, flavonol synthase activity, and positive regulation of flavonoid biosynthetic process; flavonoid-3′,5′-hydroxylase activity; isoflavone-2′-hydroxylase activity; and flavanone-4-reductase activity. The TransM12 was highly correlated with the metabolite MetM2 module, which represented the largest metabolite module and included flavonoids, coumarins, phloroglucinols, and benzopyrans. Our results showed that the WCNA module-based correlation appropriately captured the relationship between the type of metabolite being accumulated and the genes associated with its biosynthesis, further exploring which one helps narrow down the candidate genes involved in the biosynthesis of specialized metabolites in *M. japonicus*.

### 2.5. Metabolome-Assisted Identification of Genes Associated with Rutin Biosynthesis

**Figure 7:**
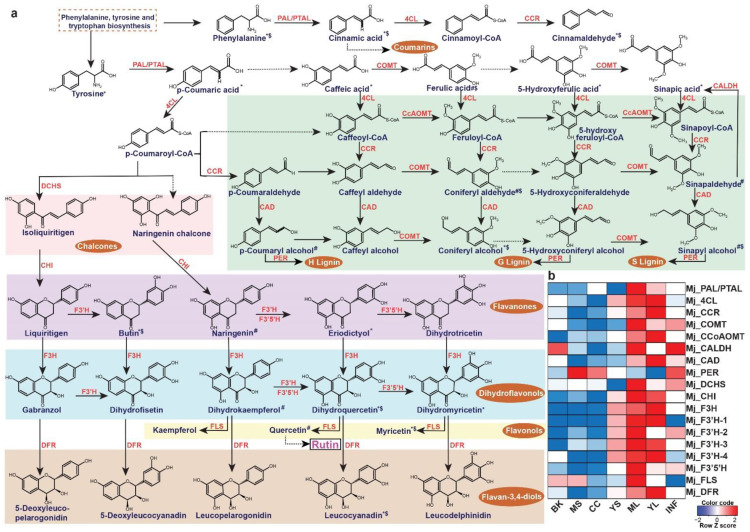
Unigenes associated with the biosynthesis of rutin and their expression levels across different tissues of *Mallotus japonicus*. (**a**) Proposed rutin biosynthetic pathway and related metabolites in *M. japonicus*. ‘*’, ‘#’, and ‘$’ represent the metabolite features mapped to the KNApSAcK database, KEGG database, and MS^2^-based fragmentation data in the *M. japonicus* metabolome database, respectively; (**b**) Heatmap representing the expression of transcripts assigned to the rutin biosynthetic pathway in *M. japonicus*. Abbreviations: PAL/PTAL: phenylalanine/tyrosine ammonia-lyase, 4CL: 4-coumarate: CoA ligase, cinnamoyl-CoA reductase, COMT: caffeic acid OMT, CcoAOMT: caffeoyl-CoA OMT, CAD: cinnamyl alcohol dehydrogenase, PER: peroxidase, DCHS: 6′-deoxychalcone synthase, CHI: chalcone isomerase, F3H: flavanone-3-hydroxylase, F3′H: flavonoid-3′-hydroxylase, F3′5′H: flavonoid-3′,5′-hydroxylase, FLS: flavonol synthase, DFR: dihydroflavonol 4-reductase. ML: Mature leaf, YS: Young stem, YL: Young leaf, CC: Central cylinder, MS: Mature stem, INF: Inflorescence, and BK: Bark.

The leaves of *M. japonicus* are known to treat boils and swellings and represent the tissue accumulating several bioactive moiety-containing metabolites [5]. Rutin, one of the most important metabolites in the leaves contributing to its medicinal properties, is a member of the MetM7 metabolite module. Moreover, metabolites assigned to the MetM7 module showed high accumulation in the leaf tissue, including young and mature leaves (Figure 4). We, therefore, hypothesized that the transcript modules correlated with the MetM7 metabolite module would include functional genes most likely associated with the biosynthesis of rutin and associated metabolites. Several studies have highlighted the importance of integrating transcriptome and metabolome datasets in identifying functional genes [29,38,54,55,56,57,58]. Therefore, using a similar strategy in *M. japonicus*, we performed a correlation analysis between metabolite and transcript modules and identified the transcript modules TransM9 and TransM4, sharing a high correlation (R^2^ > 0.7) with the MetM7 metabolite module. Interestingly, KEGG pathway mapping showed homologs for genes representing the entire phenylpropanoid and flavonoid biosynthetic pathways included in the TransM4 and TransM9 transcript modules. (Figure 7a, Figures S6 and S7 and Table S15). Moreover, the metabolite module-driven correlation analysis approach drastically eliminated potential false-positive genes assigned to the phenylpropanoid and flavonoid biosynthesis derived only from the homology-based annotation approach. Using the metabolome-guided approach, we assigned 18 transcripts to the biosynthesis of rutin and associated metabolites (Figure 7b). Subsequently, we checked the expression level of the assigned genes across the seven tissues of *M. japonicus.* Similar to the accumulation pattern of rutin, the assigned biosynthetic genes were highly expressed in the leaf tissues. Several studies across different plant species have suggested a high correlation between flavonol synthase (FLS) expression and the accumulation of rutin [59,60]. Shi et al. [61] reported a 3.5 to 4.4-fold increase in rutin accumulation level in the tobacco leaves overexpressing the *NtFLS2* gene. In contrast, no significant change in the levels of the intermediates was observed except for kaempferol-3-*O*-rutinoside, thus highlighting the role of FLS in the biosynthesis of rutin. The expression pattern of the assigned FLS gene in *M. japonicus* correlated with the accumulation pattern of rutin, which was most abundant in the mature leaves. These results suggest that the mature leaf is the active site for rutin biosynthesis in *M. japonicus*. The 18 genes identified herein are strong candidates involved in the biosynthesis of rutin in *M. japonicus*. Our results, therefore, clearly emphasizes the importance of a metabolome-guided approach in narrowing down the candidate genes involved in the biosynthesis of specialized metabolites, further functional characterization of which could ascertain their role in the biosynthetic network.

### 2.6. Phylogenetic Analysis of Glucosyltransferases and O-methyltransferases Reveals Insights into Bergenin Biosynthesis in Mallotus japonicus

**Figure 8:**
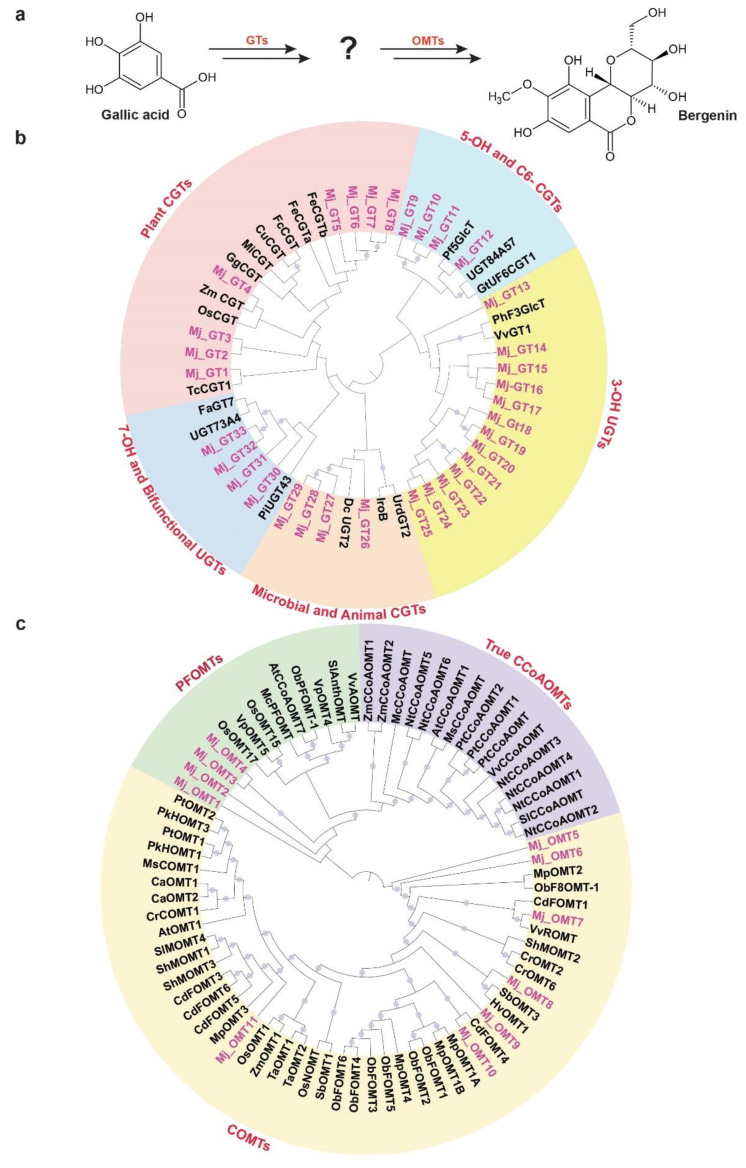
Identification of putative candidate unigenes involved in the biosynthesis of bergenin in *Mallotus japonicus*. (**a**) Proposed pathway for the conversation of gallic acid to bergenin catalyzed by glucosyltransferases and *O*-methyltransferases. Phylogenetic analysis of (**b**) glucosyltransferases (GTs) and (**c**) *O*-methyltransferases (OMTs) using *M. japonicus* candidate genes together with functionally characterized genes across diverse plant species. Thirty-three and eleven unigenes annotated as GTs and OMTs, respectively, were selected from the transcript modules that were highly correlated with the metabolite module MetM3 and MetM6, which included gallic acid and bergenin, respectively. Nucleotide sequences were translated to their corresponding protein sequences, and the alignment and phylogenetic reconstructions were performed using ETE3 v3.1 after applying 1000 replications. Bootstrap values above 60% are shown here.

Bergenin, a dihydroisocoumarin derivative, includes β-D-glucosyl residues C-linked to a hydroxylated phenyl carboxylic acid ortho to the carboxyl group. Additionally, the carboxyl group is esterified with the C-2 hydroxyl group of the glucosyl moiety to form a δ-lactone ring [62]. Bergenin represents one of the most important medicinal compounds in *M. japonicus* [1,2]. Metabolite profiling for multiple tissues of *M. japonicus* showed the highest accumulation of bergenin and related metabolites in the bark tissue, followed by the mature stem (Figure 4), which explains bark being the primary tissue used for various medicinal purposes [1,2]. While little is known about the metabolite intermediates and enzymes involved in the biosynthesis of bergenin, Tateyama and Yoshida [63] proposed gallic acid as the precursor for the bergenin biosynthesis based on their labeled experimental approach in *Saxifraga stolonifera*. Gallic acid acts as the glucosyl acceptor and undergoes C-glycosylation, which subsequently undergoes methylation to form bergenin (Figure 8a). WCNA analysis for MS^2^-validated metabolites assigned gallic acid and bergenin to the MetM3 and MetM6 metabolite modules, respectively. To identify potential candidate glucosyltransferases (GTs) and *O*-methyltransferases (OMTs) coding genes that may catalyze the C-glycosylation and methylation reactions resulting in the biosynthesis of bergenin from gallic acid, we focused on the TransM8, TransM10, and TransM15 transcript modules, which were highly correlated with the MetM3 and MetM6 metabolite modules (Figure 6a). In total, these three transcript modules included 33 GTs and 11 OMTs (Figure S8 and Table S16), which were further used for phylogenetic analysis.

Phylogenetic analysis for 33 GTs was performed with 20 functionally characterized GTs representing a diverse family of GTs from different species (Table S17). Of the 33 GTs of *M. japonicus*, 13 were classified together, with the GTs catalyzing 3-OH glycosylation, while four GTs each were grouped with GT families catalyzing C-glycosylation in microbial and animal systems, and 7-OH glycosylation, respectively (Figure 8b). C6-GTs and 5-OH GTs are closely related gene families, and a mutual exchange of the two amino acid residues has been reported to switch the O- and C-glycosylation activity [64]. Four GT-annotated genes from *M. japonicus* were grouped with GTs catalyzing the C6-glycosylation and 5-OH glycosylation of flavonoids, suggesting a potential role in deriving metabo-diversity. Eight GT genes from *M. japonicus* were phylogenetically related with GTs known to catalyze C-glycosylation in different plant species (Figure 8b). Among these, Mj_GT1 was phylogenetically related to TcCGT1, known to catalyze the C-glycosylation of 83 substrates, including 36 different structural types of flavonoids, and benzophenones, which is similar to gallic acid, belongs to the benzenoid class of metabolites [65]. Another gene, Mj_GT4, was closely related to Zm_CGT and Os_CGT. Os_CGT, apart from its natural substrates, has also been shown to catalyze the C-glycosylation of several synthetic 2-hydroxyflavanones, which share structural similarity with gallic acid in terms of containing substituted benzene rings [66,67,68]. Mj_GT1 and Mj_GT4, sharing close homology and phylogeny with the functionally characterized GTs demonstrating a wide range of substrate and high expressions in young stem and bark tissues, respectively, have potential roles in catalyzing the C-glycosylation of gallic acid towards the biosynthesis of bergenin. Therefore, these two genes are assigned here as potential enzymes that catalyze gallic acid to drive bergenin or bergenin-like metabolite biosynthesis in plants.

In the later step of bergenin biosynthesis, the C-glycosylated form of gallic acid is subsequently methylated, which is catalyzed by the O-methyltransferase (OMT) family of enzymes (Figure 8a) [63]. In plants, OMTs are divided into two major families: caffeic acid OMTs (COMTs) and caffeoyl-CoA OMTs (CCoAOMTs) [69]. COMTs participate in the methylation of flavonoids, while CCoAOMTs catalyze lignin biosynthesis by acting on phenylpropanoid-CoA esters [70]. Several of the CCoAOMTs from different plant species have also been recently reported to methylate a wide range of metabolites, and hence, they have been further classified into a new subgroup: PFOMT [71,72,73]. To identify the potential candidate methyltransferase enzymes that may catalyze the methylation step towards the biosynthesis of bergenin, we performed a phylogenetic analysis using functionally characterized methyltransferases with 11 OMTs identified in *M. japonicus* transcript modules being highly correlated with metabolite modules consisting of gallic acid and bergenin (Figure 6a and Table S16). The phylogenetic analysis showed the genes separated into two major clades: clade 1 containing COMTs, while clade 2 included CCoAOMTs subdivided into true CCoAOMTs and PFOMTs (Figure 8c). Seven of the OMTs from *M. japonicus* were grouped in clade 1 with other known COMTs, while four were clustered together with clade 2, specifically with PFOMTs. Of the four OMTs in the PFOMTs clade, three were members of the transcript module TransM8, and one of them was a member of the TransM10 transcript module. While the function of additional PFOMTs (CCoAOMTs-like) has not been characterized extensively in plants, the OMTs from *M. japonicus* being grouped with functionally characterized PFOMTs were reported to have specificity for a wide variety of substrates. Its high correlation with either the precursor, gallic acid, or the final product, bergenin, might indicate its potential role in the biosynthesis of bergenin in *M. japonicus*. The integrative omics-based strategy helped us identify the potential GTs and OMTs involved in the biosynthesis of bergenin. By exploring the established metabolome and transcriptome resource, we identified potential candidate genes involved in the biosynthesis of specialized metabolites imparting medicinal properties to different tissues of *M. japonicus*. Their subsequent functional characterization in future studies will help ascertain their role in the bergenin biosynthesis in *M. japonicus*. Our results showed the quality of omics resources established in this study, which will be valuable in understanding the mechanisms and components that associate specific medicinal properties to different tissues of *M. japonicus*.

## 3. Materials and Methods

### 3.1. Plant Materials

The *Mallotus japonicus* tree was maintained at the medicinal plant gardens, Chiba University, under natural conditions (voucher number: MJ_CU001, Figure S9). The seven tissues of *M. japonicus*, including young leaf, mature leaf, young stem, mature stem, bark, central cylinder, and inflorescence, were harvested and immediately frozen in liquid nitrogen. The tissues were stored at −80 °C until further use.

### 3.2. Untargeted Metabolite Profiling Using LC-QTOF-MS

The tissue samples were freeze-dried using a freeze dryer, FDU-2200 (Tokyo Rikakikai CO., Ltd., Tokyo, Japan), and were subsequently used for metabolite extraction using 50 μL of 80% (*v*/*v*) LC–MS-grade methanol (Wako chemicals, Osaka, Japan) and 20% (*v*/*v*) LC–MS-grade water (Wako chemicals, Osaka, Japan) containing 2.5 μM of lidocaine (TCI, Tokyo, Japan) and 10-camphoursulfonic acid (TCI, Tokyo, Japan) per milligram of dry weight. The mixture was homogenized using a mixer mill, MM300 (Retsch, Haan, Germany), with a zirconia bead at 18 Hz at 4 °C for 7 min. Furthermore, it was centrifuged at 17,800× *g* for 10 min, and the supernatant was filtered using Oasis HLB μElution plate (Waters Inc., Milford, MA, USA). The MS and MS^2^ datasets were acquired as described previously [38]. In brief, 1 μL of the extracts was analyzed using LC–QTOF–MS (LC, Waters Acquity UPLC system, Waters, MA, USA); MS, Waters Xevo G2 Q-Tof, Waters, MA, USA). The analytical conditions were as follows: LC: column, Acquity bridged ethyl hybrid C18 (1.7 μm, 2.1 mm × 100 mm, Waters); solvent system, solvent A (water including 0.1% (*v*/*v*) formic acid) and solvent B (acetonitrile including 0.1% (*v*/*v*) formic acid); gradient program, 99.5%A/0.5%B at 0 min, 99.5%A/0.5%B at 0.1 min, 20%A/80%B at 10 min, 0.5%A/99.5%B at 10.1 min, 0.5%A/99.5%B at 12.0 min, 99.5%A/0.5%B at 12.1 min, and 99.5%A/0.5%B at 15.0 min; flow rate, 0.3 mL/min at 0 min, 0.3 mL/min at 10 min, 0.4 mL/min at 10.1 min, 0.4 mL/min at 14.4 min, and 0.3 mL/min at 14.5 min; column temperature, 40 °C; MS detection: capillary voltage, +3.00 kV (positive)/−2.75 kV (negative); cone voltage, 25.0 V; source temperature, 120 °C; desolvation temperature, 450 °C; cone gas flow, 50 L/h; desolvation gas flow, 800 L/h; collision energy, 6 V; mass range, *m*/*z* 100‒1500; scan duration, 0.1 s; inter-scan delay, 0.014 s; data acquisition, centroid mode; polarity, positive/negative: scan duration, 1.0 s; and inter-scan delay, 0.1 s. THe MS^2^ data were acquired in the ramp mode as the following analytical conditions: (1) MS: mass range, *m*/*z* 50–1500; scan duration, 0.1 s; inter-scan delay, 0.014 s; data acquisition, centroid mode; and polarity, positive/negative and (2) MS^2^: mass range, *m/z* 50–1500; scan duration, 0.02 s; inter-scan delay, 0.014 s; data acquisition, centroid mode; polarity, positive/negative; and collision energy, ramped from 10 to 50 V. The MS^2^ spectra of the top 10 ions, with counts over 1000 in the MS scan, were acquired and moved further to the next top 10 ions based on MS scans at a given run time, while the acquisition was not performed if the ion intensity was <1000. The data were processed using MS-DIAL with default parameters, and the acquired metabolite peaks were used for Principal Component Analysis (PCA). The MS^2^-based validation of metabolite identity was performed as described previously [38]. The average level for the five bio replicates of these MS^2^ validated metabolites was used further for the identification of metabolite modules using the WGCNA package in R [23].

### 3.3. Feature-Based Molecular Networking

A molecular network was created with the Feature-Based Molecular Networking (FBMN) workflow on GNPS (https://gnps.ucsd.edu, accessed on 30 November 2020). The mass spectrometry data were first processed with MS-DIAL, and the results were exported to GNPS for FBMN analysis. The data were filtered by removing all of the MS^2^ fragment ions within +/−17 Da of the precursor *m*/*z*. The MS/MS spectra were window filtered by choosing only the top six fragment ions in the +/−50 Da window throughout the spectrum. The precursor ion mass tolerance was set to 0.02 Da, and the MS/MS fragment ion tolerance to 0.02 Da. A molecular network was then created, where edges were filtered with a cosine score above 0.5 and more than three matched peaks. Furthermore, edges between two nodes were kept in the network if and only if each of the nodes appeared in each other’s respective top 10 most similar nodes. Finally, the maximum size of a molecular family was set to 50, and the lowest-scoring edges were removed from molecular families until the molecular family size was below this threshold. The analogue search mode was used by searching against MS^2^ spectra with a maximum difference of 100.0 in the precursor ion value. The library spectra were filtered in the same manner as the input data. All matches were kept between network spectra, and library spectra were required to have a score above 0.5 and at least three matched peaks. The DEREPLICATOR was used to annotate MS/MS spectra [74]. The molecular networks were visualized using *Cytoscape* software v3.8. [75].

### 3.4. RNA Isolation and cDNA Synthesis

The tissues were homogenized for RNA extraction. Total RNA was extracted from all seven tissues as described previously [76]. RNA with RNA integrity number (RIN) over 8.0 were further used to synthesize cDNA libraries, as described previously [76].

### 3.5. RNA-Sequencing and Processing of Raw Data to Generate Transcriptome Assembly

The cDNA libraries for seven tissues of *M. japonicus* were sequenced using Illumina HiSeq™ 2000 (Illumina Inc., San Diego, CA, USA) to obtain a paired-end read with an average read-length of 101 bps. The construction of cDNA libraries and their sequencing were performed at Kazusa DNA Research Institute, Japan. The raw sequence reads were then processed to remove adaptor sequences, low-quality reads, short-read, and ambiguous read sequences using the Trimmomatic program. The high-quality reads were subsequently used to generate de novo transcriptome assembly.

The de novo transcriptome assembly was generated using three popular assemblers; namely, CLC genomics workbench (https://www.qiagenbioinformatics.com/, accessed on 8 May 2020), Trinity with default parameters, and for the SOAPdenovo-Trans, six different k-mer sizes were used, namely, 31, 41, 51, 63, 71, and 91. The transcriptome assembly of SOAPdenovo-Trans with k-mer 31 was best compared with other used k-mers and, therefore, was selected for its concatenation with individual assembly derived from Trinity and CLC genomics workbench (Table 1). The concatenated transcriptome assembly was further processed using CD-HIT-EST [40] for removing the sequence redundancy, if any, as described previously [77]. We evaluated the completeness and quality of the *M. japonicus* de novo transcriptome assembly based on Benchmarking Universal Single-Copy Orthologs (BUSCO v.5.1.0) analysis using embryophyta_odb10 database [39].

### 3.6. Functional Classification, KEGG Pathway Mapping, and Expression Analysis of the Assembled Transcripts of Mallotus japonicus

A Blast-x based homology search was performed as previously described for the annotation of *M. japonicus* de novo transcriptome assembly. The resulting top-blast hit was used for the annotation of the de novo transcriptome assembly. Furthermore, OmicsBox software v2.0 (https://www.biobam.com/, accessed on 31 March 2021) was used to retrieve the associated EC number, GO terms, and KEGG pathway-based annotation.

The expression values of the assembled transcripts were estimated in terms of Fragments Per Kilobase of transcript per Million mapped reads (FPKM) values as described elsewhere [77].

### 3.7. Module Construction Using Transcriptome Dataset

For the identification of modules in the transcriptome datasets, we first filtered the assembled transcripts using the following parameters: (i) length > 500 bps and similarity > 70%, and (ii) FPKM > 5 in at least one of the seven tissues. Based on the above two criteria, we narrowed down 30,497 transcripts, which were subsequently used for WGCNA analysis in R. Hierarchical clustering based on the topological overlap (TO) similarity was used to obtain network modules with a minimum of 100 transcripts, and the highly similar modules (dissimilarity < 0.25) were merged to prevent over-splitting of the modules. Module eigenvalues, representing the overall profile of the module, were calculated for each of the identified modules.

### 3.8. Correlation Calculation between the Identified Metabolite and Transcript Modules

The averaged FPKM values and metabolite levels clustered in the individual metabolite and transcript modules were used for correlation analysis. A Pearson correlation coefficient was calculated between each metabolite and transcript module to generate a pairwise Pearson correlation matrix, which was subsequently visualized using the Heatmap2.0 package in R.

### 3.9. Gene Ontology Enrichment Analysis

The Gene Ontology (GO) enrichment analysis was performed using OmicsBox software using the transcripts clustered in individual modules as a test set and the entire transcriptome assembly as a reference set. Furthermore, GO terms with a corrected *p*-value < 0.01 were considered significantly enriched.

### 3.10. Phylogenetic Analysis of GTs and O-Methyltransferases

The selected transcripts of *M. japonicus* encoding GTs and OMTs were translated to their corresponding protein sequences using OmicBox software v2.0 (https://www.biobam.com/, accessed on 31 March 2021). Furthermore, the corresponding protein sequences were combined with the known GTs and OMTs from the databases. The alignment and phylogenetic reconstructions were performed using the function “build” of ETE3 v3.1.1 [78] as implemented on the GenomeNet (https://www.genome.jp/tools/ete/, accessed on 30 November 2020). Alignment was performed with MUSCLE v3.8.31 with the default options [79]. The resulting alignment was cleaned using the gappyout algorithm of trimAl v1.4.rev6 [80]. The best protein model was selected using ML tree inference among the JTT, WAG, VT, LG, and MtREV models using pmodeltest v1.4. The ML tree was inferred using RAxML v8.1.20 run with model PROTGAMMALGF and default parameters [81]. Branch supports were computed out of 1000 bootstrapped trees. The phylogenetic tree was visualized and customized using the online tool iTOL [82].

## 4. Conclusions

Traditional medicine still constitutes one of the effective means in alleviating human diseases. While cataloging plant species and specific tissue types for medical purposes based on human experiences has been the basis of traditional medicine practices for thousands of years, the advent of modern analytical tools has begun to elucidate specific phytochemicals contributing to its unique medicinal properties. Advances in generating multi-omics datasets and analysis pipelines are valuable in identifying molecular players that contribute to the biosynthesis and regulation of important bioactive metabolites. Such resources are key to exploring and understanding the biosynthesis of specialized metabolites, including identifying novel bioactive metabolites, and serve as the basis to meet sustainable development goals for a healthy society. To establish high-quality genomics and metabolomics resources for the valuable medicinal plant, *M. japonicus*, we performed deep RNA-seq based transcriptome profiling and untargeted metabolite profiling for multiple tissues, including tissues used in the traditional medicinal practices.

In total, we identified 2129 metabolites by mapping the mass features to the KNApSAcK database. The feature-based molecular networking helped us identify the overall metabo-classes present across seven tissues of *M. japonicus*. Furthermore, we validated the identity of 69 metabolites via their MS^2^-based fragmentation pattern, and their accumulation trend across seven tissues showed different tissues of *M. japonicus* with specific metabolic signatures consistent with their medicinal properties. We further established a de novo transcriptome assembly of *M. japonicus* using multiple tissues to capture diverse and active transcripts-state associated with tissue-specific metabolites biosynthesis. The module-based integration of transcriptome and metabolome datasets followed by phylogenetic analysis helped us identify potential candidate genes involved in the biosynthesis of specialized metabolites, namely rutin and bergenin, in *M. japonicus*. Our results reconfirmed the tissue specificity of specialized metabolites biosynthesis as observed in the case of *M. japonicus*. The metabolome and transcriptome resources being established in this study serves as a stepping-stone for identifying novel metabolites with pharmacological importance present in *M. japonicus* and their associated biosynthetic pathway through functional characterization of associated transcripts discovered in this study.

## Figures and Tables

**Table 1:** Assembly statistics for *Mallotus japonicus* de novo transcriptome assembly based on three popular assemblers and their combination.

Assembler	Kmer	No. of Contigs	N50	Mean Length	Median Length	Max Length	*n*: >500	*n*: >1000	Total Size of Contigs
**CLC**	20	108,863	769	592	366	17,150	35,538 (32.6%)	14,771 (13.6%)	64,424,765
**Trinity**	25	321,190	1503	879	486	17,097	156,853 (48.8%)	90,563 (28.2%)	282,445,544
**SOAPdenovo**	31	278,339	595	322	156	17,202	34,941 (12.6%)	17,967 (6.5%)	89,684,117
	41	276,091	525	325	168	17,225	34,755 (12.6%)	17,259 (6.3%)	89,843,229
	51	337,219	373	278	160	17,210	33,450 (9.9%)	15,902 (4.7%)	93,877,046
	63	273,737	397	307	181	17,292	31,027 (11.3%)	14,829 (5.4%)	84,133,522
	71	209,952	484	345	199	16,558	28,293 (13.5%)	14,006 (6.7%)	72,338,185
	91	46,669	1062	585	294	11,896	15,185 (32.5%)	8137 (17.4%)	27,278,317
**CLC_Trinity_SOAPdenovo (kmer31) _CD-HIT-EST**	N.A.	226,250	1396	837	469	17,202	106,994 (47.3%)	58,421 (25.8%)	189,317,381

## Data Availability

The raw sequence reads for all seven tissues of *M. japonicus*, the expression values, and the de novo transcriptome assembly used in this study have been deposited in NCBI’s Gene Expression Omnibus (GEO) and are available at the GEO accession number GSE179338.

## Supplementary Materials

The following are available online at https://www.mdpi.com/article/10.3390/ijms22168835/s1.
