# Supplementary material for: Gene-Metabolite Network Analysis Revealed Tissue-Specific Accumulation of Therapeutic Metabolites in Mallotus japonicus
Source: Int J Mol Sci. 2021 Aug 17;22(16):8835. doi: 10.3390/ijms22168835 (PMC8396295; doi:10.3390/ijms22168835)
Supplement: Supplementary file 1 [file ijms-22-08835-s001.zip › Figure S1 Features of de novo transcriptome assembly of Mallotus japonicus with legend.pdf]

a

b

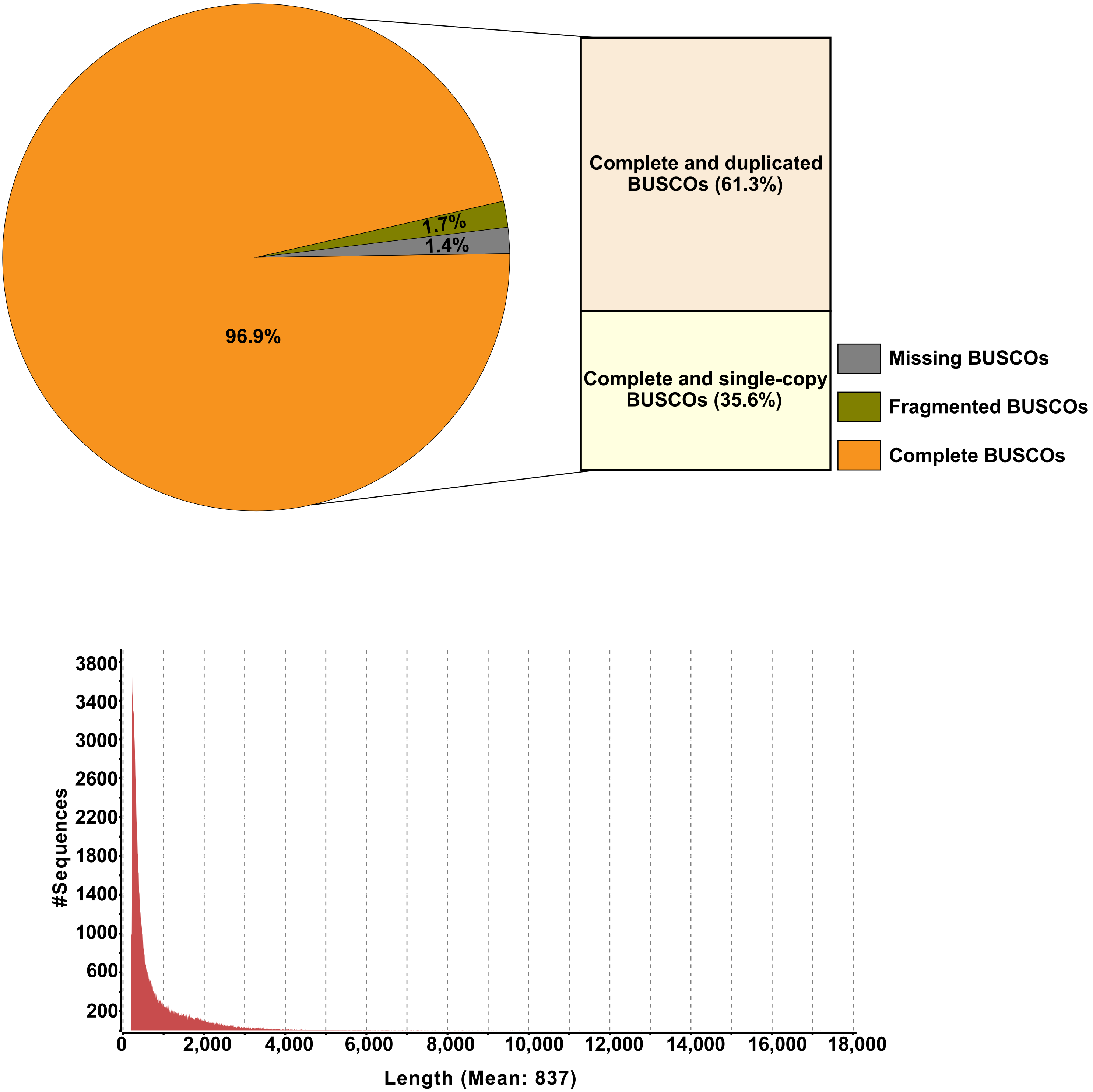

Figure S1. Features of *de novo* transcriptome assembly of *Mallotus japonicus.* (a) Evaluation of *M. japonicus de novo* transcriptome assembly using Benchmarking Universal Single-copy Orthologs (BUSCO) analysis; **(b)** Transcript length distribution plot for *de novo* transcriptome assembly of *M. japonicus*.
