# Supplementary material for: Gene-Metabolite Network Analysis Revealed Tissue-Specific Accumulation of Therapeutic Metabolites in Mallotus japonicus
Source: Int J Mol Sci. 2021 Aug 17;22(16):8835. doi: 10.3390/ijms22168835 (PMC8396295; doi:10.3390/ijms22168835)
Supplement: Supplementary file 1 [file ijms-22-08835-s001.zip › Figure S2 Functional characterization of the assembled de novo transcriptome of Mallotus japonicus with legend.pdf]

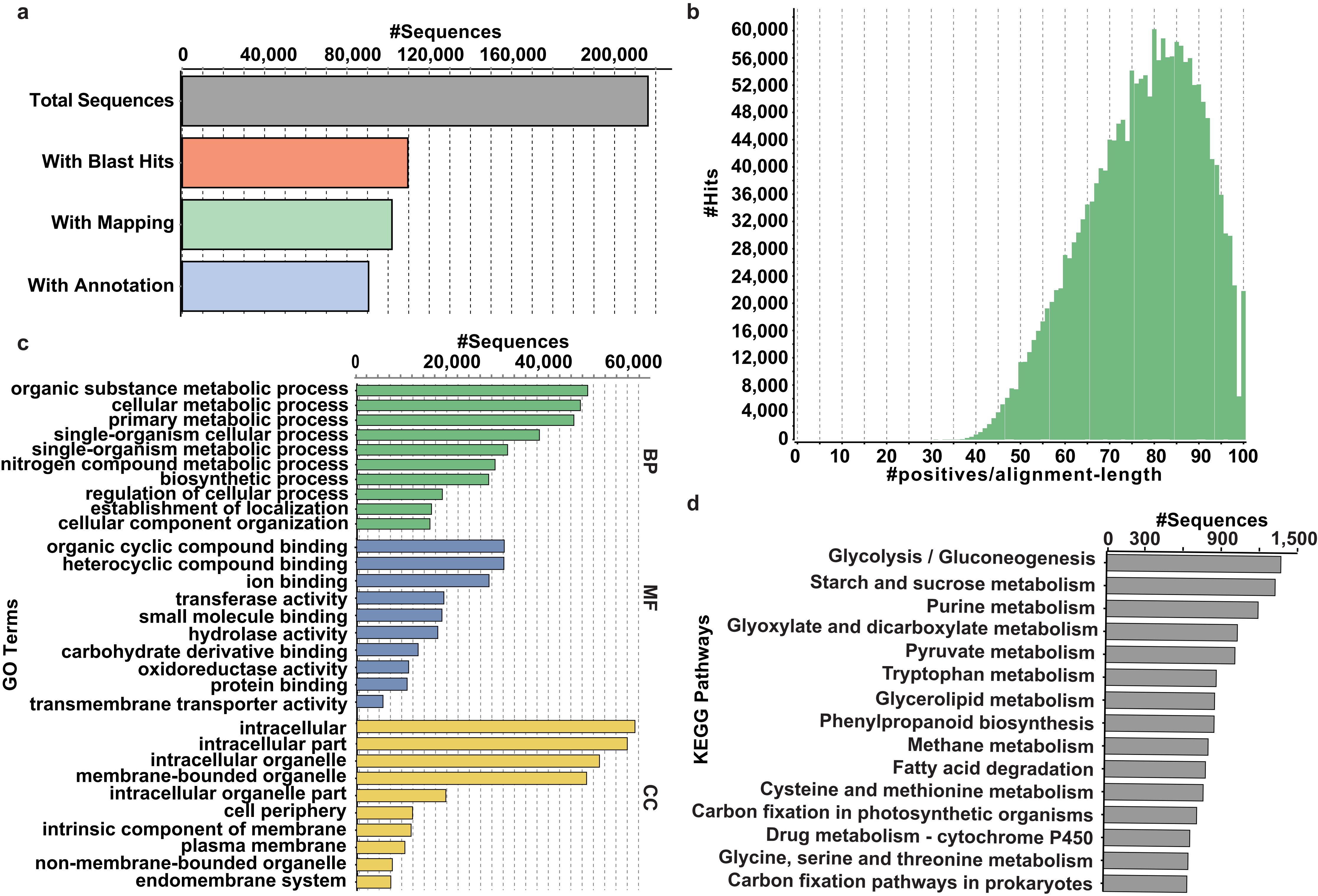


**Figure S2.** Functional classification of the *de novo* transcriptome assembly of *Mallotus japonicus*. **(a)** Homology based annotation summary for the assembled transcripts of *M. japonicus*; **(b)** Sequence-similarity distribution plot for the annotated transcripts using top blastx hits; **(c)** Gene ontology (GO) distribution plot for *M. japonicus* transcriptome assembly. GO terms for the annotated transcripts are summarized in three functional categories: biological processes (BP), molecular function (MF), and cellular component (CC); **(d)** Top 15 KEGG pathways represented by the assembled transcripts of *M. japonicus* based on the number of assigned transcripts.
