# Supplementary material for: Gene-Metabolite Network Analysis Revealed Tissue-Specific Accumulation of Therapeutic Metabolites in Mallotus japonicus
Source: Int J Mol Sci. 2021 Aug 17;22(16):8835. doi: 10.3390/ijms22168835 (PMC8396295; doi:10.3390/ijms22168835)
Supplement: Supplementary file 1 [file ijms-22-08835-s001.zip › Figure S3 Transcriptome expression analysis across seven tissues of Mallotus japonicus with legend.pdf]

a

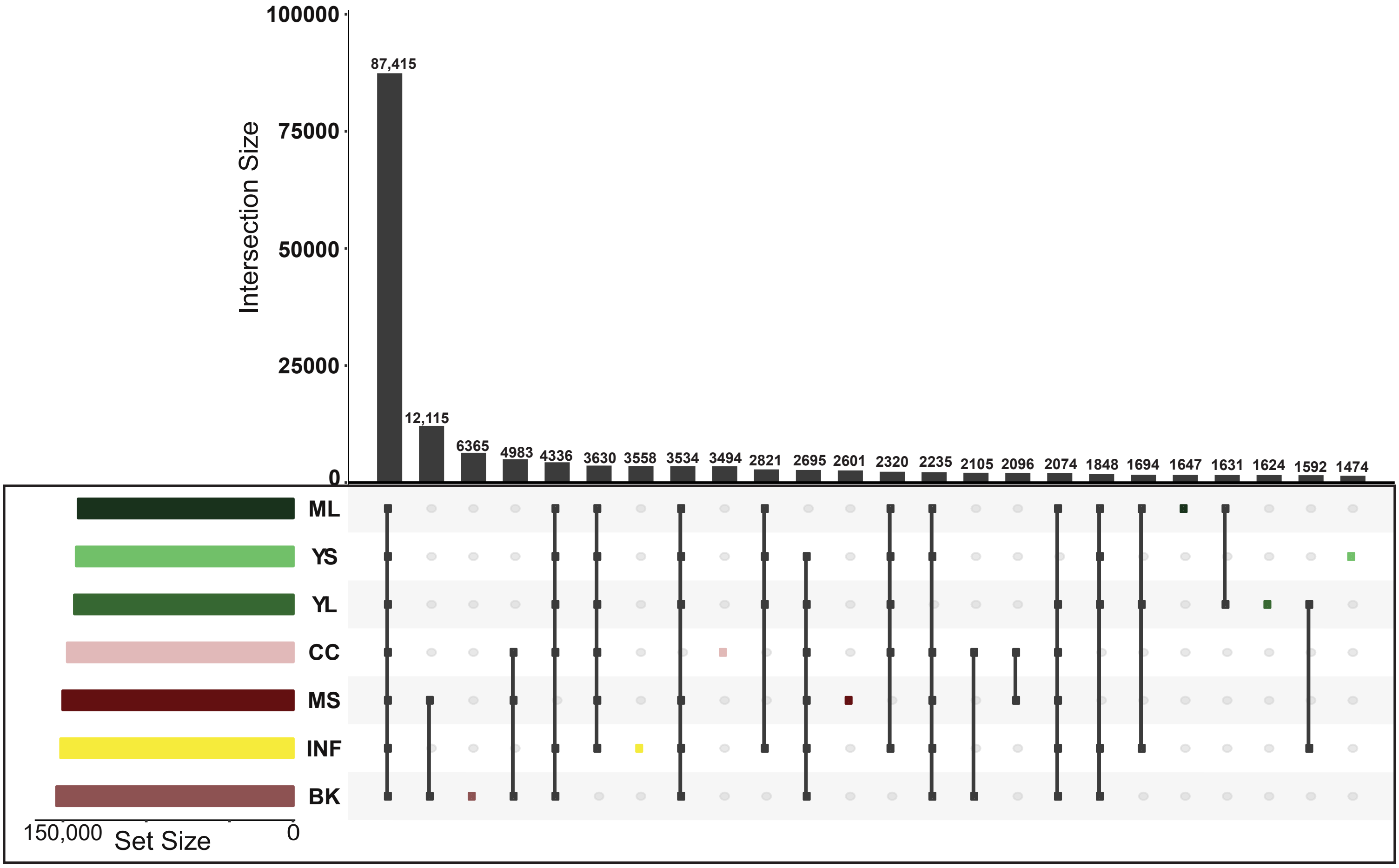

b

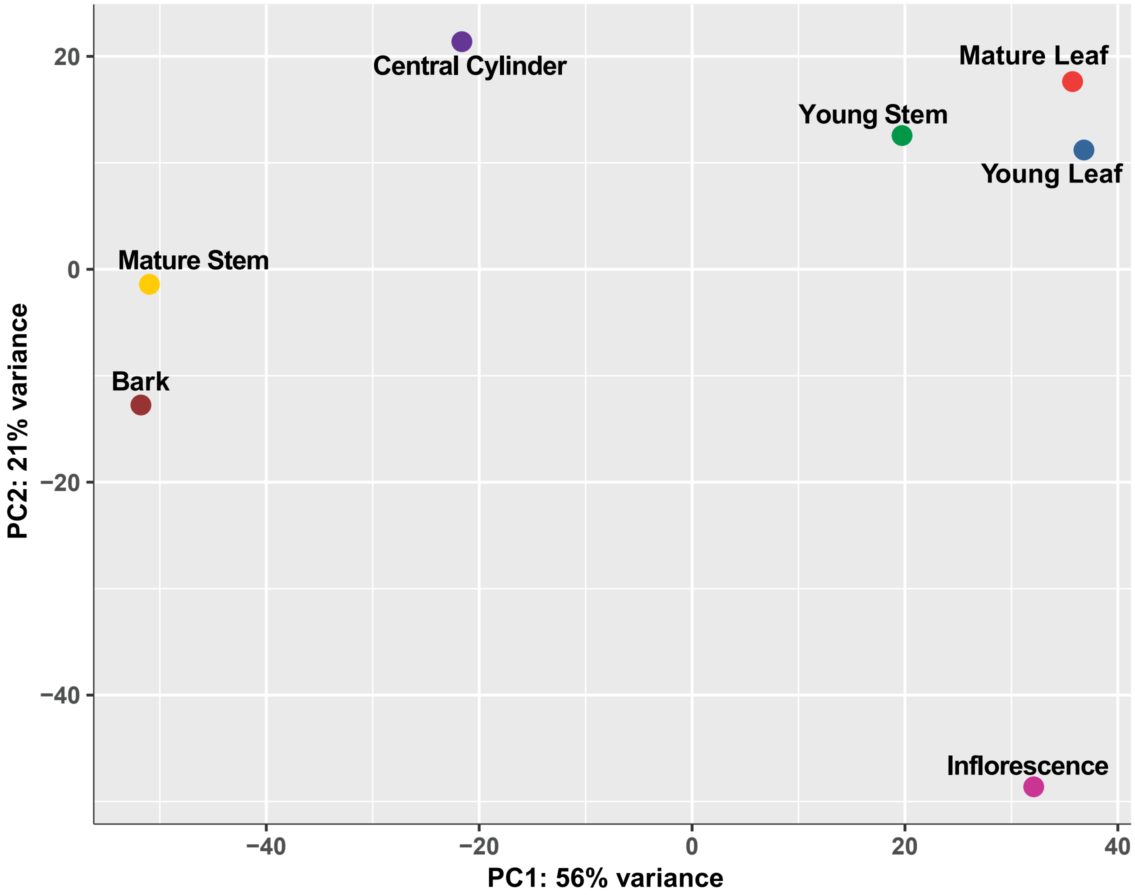

Figure S3. Transcriptome expression analysis across seven tissues of *Mallotus japonicus*. **(a)** Upset plot-based representation for the number of shared and unique active transcripts across each of the tissue of *M. japonicus*. The overlap of transcripts over 1,450 is being showed here. Abbreviations: ML: Mature leaf, YS: Young stem, YL: Young leaf, CC: Central cylinder, MS: Mature stem, INF: Inflorescence, and BK: Bark; **(b)** Unsupervised principal component analysis for transcripts expression across seven tissues of *M. japonicus*.
