# Supplementary material for: Gene-Metabolite Network Analysis Revealed Tissue-Specific Accumulation of Therapeutic Metabolites in Mallotus japonicus
Source: Int J Mol Sci. 2021 Aug 17;22(16):8835. doi: 10.3390/ijms22168835 (PMC8396295; doi:10.3390/ijms22168835)
Supplement: Supplementary file 1 [file ijms-22-08835-s001.zip › Figure S4 Expression levels of transcripts included in each TransM module across seven tissues of Mallotus japonicus with legend_Megha.pdf]

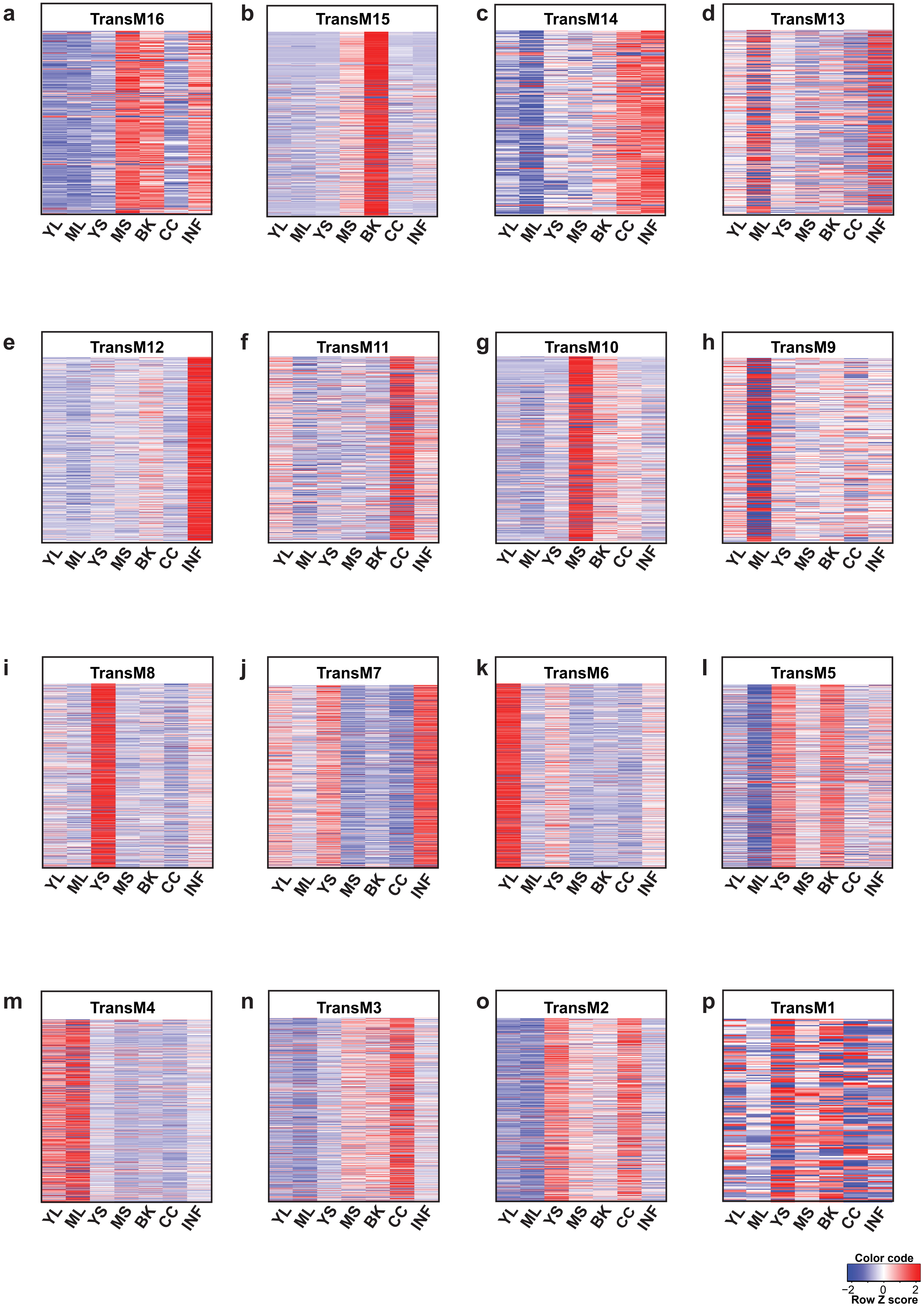

Figure S4 Expression levels of transcripts included in each TransM module across seven tissues of *Mallotus japonicus*
