# Supplementary material for: Gene-Metabolite Network Analysis Revealed Tissue-Specific Accumulation of Therapeutic Metabolites in Mallotus japonicus
Source: Int J Mol Sci. 2021 Aug 17;22(16):8835. doi: 10.3390/ijms22168835 (PMC8396295; doi:10.3390/ijms22168835)
Supplement: Supplementary file 1 [file ijms-22-08835-s001.zip › Figure S8 Expression profile for GTs and OMTs involved in the biosynthesis of bergenin with legend.pdf]

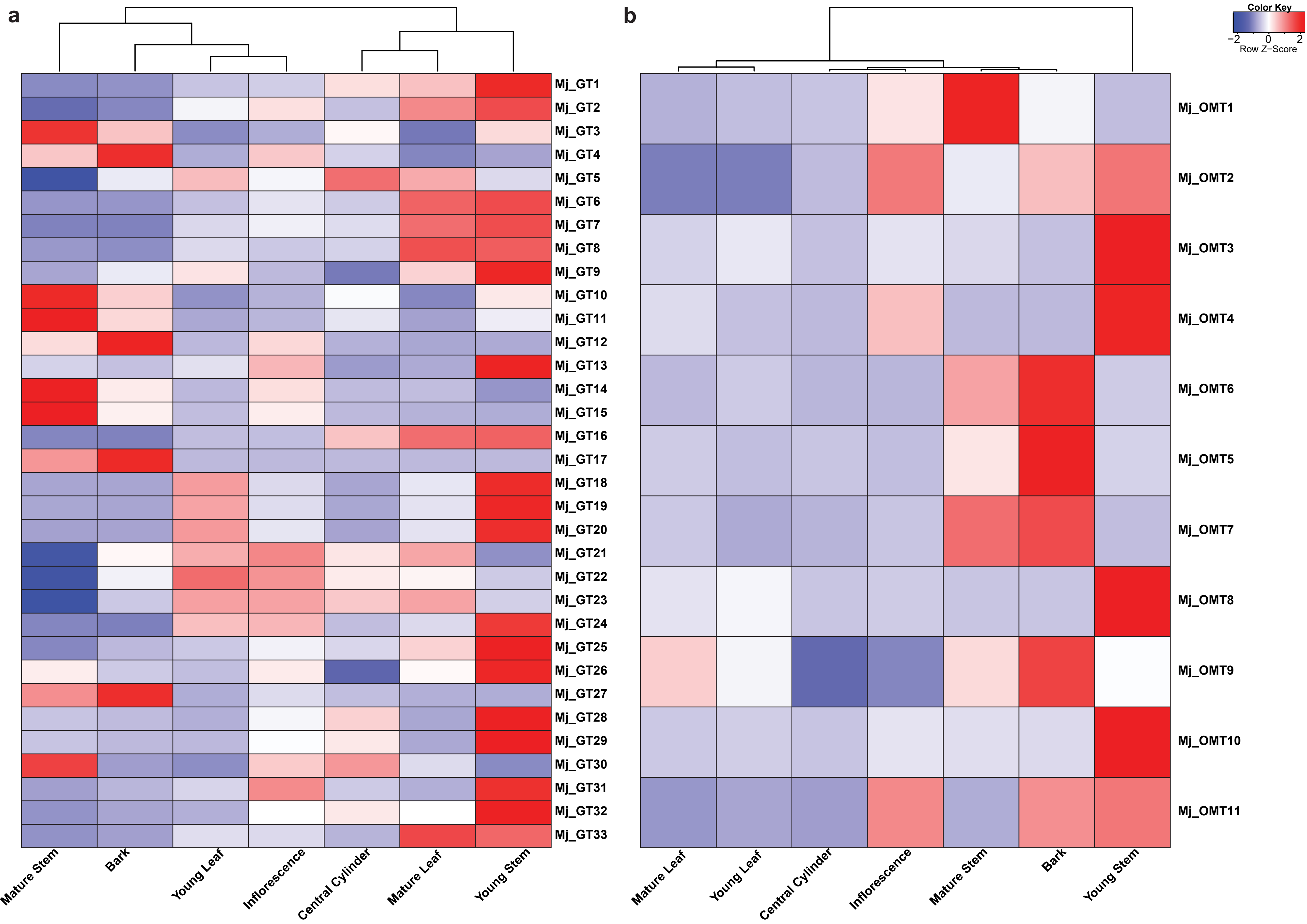


**Figure S8.** Expression profile for **(a)** candidate glucosyltransferases (GTs) and **(b)** O-methyltransferases (OMTs) involved in the biosynthesis of bergenin in *Mallotus japonicus*. Transcripts annotated as GTs or OMTs, and clustered in the transcript module sharing high correlation with metabolite module including gallic acid (MetM3) or bergenin (MetM6), were selected, and heatmap was generated using their expression value across seven tissues in R.
