# Supplementary material for: Gene-Metabolite Network Analysis Revealed Tissue-Specific Accumulation of Therapeutic Metabolites in Mallotus japonicus
Source: Int J Mol Sci. 2021 Aug 17;22(16):8835. doi: 10.3390/ijms22168835 (PMC8396295; doi:10.3390/ijms22168835)
Supplement: Supplementary file 1 [file ijms-22-08835-s001.zip › Supplementary Material Information-revised.pdf]

Supplementary Material Information

Supplementary Figure legends

Figure S1. Features of *de novo* transcriptome assembly of *Mallotus japonicus*. **(a)** Evaluation of *M. japonicus de novo* transcriptome assembly using Benchmarking Universal Single-copy Orthologs (BUSCO) analysis; **(b)** Transcript length distribution plot for *de novo* transcriptome assembly of *M. japonicus*.

Figure S2. Functional classification of the *de novo* transcriptome assembly of *Mallotus japonicus.* **(a)** Homology based annotation summary for the assembled transcripts of *M. japonicus*; **(b)** Sequence-similarity distribution plot for annotated transcripts using the top blastx hits; **(c)** Gene ontology (GO) distribution plot for *M. japonicus* transcriptome assembly. GO terms for the annotated transcripts are summarized in three functional categories: biological processes (BP), molecular function (MF), and cellular component (CC); **(d)** Top 15 KEGG pathways represented by the assembled transcripts of *M. japonicus* based on the number of assigned transcripts.

Figure S3. Transcriptome expression analysis across seven tissues of *Mallotus japonicus*. **(a)** Upset plot-based representation for the number of shared and unique active transcripts across each of the tissue of *M. japonicus*. The overlap of transcripts over 1,450 is being showed here. Abbreviations: ML: Mature leaf, YS: Young stem, YL: Young leaf, CC: Central cylinder, MS: Mature stem, INF: Inflorescence, and BK: Bark; **(b)** Unsupervised principal component analysis for transcripts expression across seven tissues of *M. japonicus*.

Figure S4. Expression levels of transcripts included in each TransM module across seven tissues of *Mallotus japonicus*.

Figure S5. Integrated network of correlated transcript and metabolite modules identified in *Mallotus japonicus*. The red circle and blue diamond represent the highly correlated (PCC > 0.7) transcript and metabolite modules, respectively. The metabolites included in the associated modules are also shown. Edges were drawn for the top three enriched GO terms (corrected p-value < 0.01, OmicsBox) from each of the three categories; biological process (shown in yellow), cellular component (shown in green), and molecular function (shown in pink) for all the highly correlated transcript modules. The network was visualized using the Cytoscape software (v. 3.7.2).

Figure S6. Representation of phenylpropanoid biosynthesis pathway by the transcripts grouped in TransM4 and TransM9 transcript modules, showing high correlation with metabolite module MetM7. The transcripts grouped in the TransM4 and TransM9 modules were filtered and KEGG pathway mapping was performed using OmicsBox software.

Figure S7. Representation of flavonoid biosynthesis pathway by the transcripts grouped in TransM4 and TransM9 transcript modules, showing high correlation with metabolite module MetM7. The transcripts grouped in the TransM4 and TransM9 modules were filtered and KEGG pathway mapping was performed using OmicsBox software.

Figure S8. Expression profile for **(a)** candidate glucosyltransferases (GTs) and **(b)** *O*-methyltransferases (OMTs) involved in the biosynthesis of bergenin in *Mallotus japonicus.* Transcripts annotated as GTs or OMTs, and clustered in the transcript module sharing high correlation with metabolite module including gallic acid (MetM3) or bergenin (MetM6), were selected, and heatmap was generated using their expression value across seven tissues in R.

Figure S9. *Mallotus japonicus* plant used in this study growing in the Chiba University Medicinal garden.

Supplementary Table legends

Table S1. Comprehensive list of total mass-features being identified in this study and their intensity level across five biological replicates of seven tissues in *Mallotus japonicus*.

Table S2. KNApSAcK and KEGG pathway mapping of the acquired mass-features.

Table S3. Feature-based molecular networking (FBMN) for the acquired metabolome dataset. The aligned mass list file generated from the MS-DIAL was used as an input for FBMN analysis using the online GNPs platform (http://gnps.ucsd.edu//).

Table S4. MS/MS fragmentation pattern of MS^2 confirmed 69 metabolites. Columns in the table includes metabolite ID, *m/z* value, retention time, daughter ions, and intensity value across five biological replicates of the seven tissues.

Table S5. Summary of metabolite modules and the included metabolites using WCNA analysis for MS^2 confirmed metabolites in *Mallotus japonicus*.

Table S6. Summary of Trimmomatic output for *Mallotus japonicus.*

Table S7. Blastx based annotation result for all the assembled transcripts of *Mallotus. japonicus.*

Table S8. A comprehensive list of KEGG pathway being represented by the transcriptome of *Mallotus japonicus* and the number of unigenes being assigned to them.

Table S9. FPKM values of all the assembled transcripts of *Mallotus japonicus.*

Table S10. Highly expressed transcripts of *Mallotus japonicus* used for WCNA analysis.

Table S11. Summary of transcript modules and the included transcripts using WCNA analysis for highly expressed transcripts in *Mallotus japonicus*.

Table S12. Pearson correlation coefficient (PCC) between each transcript and metabolite modules identified in *Mallotus japonicus*.

Table S13. KEGG pathway mapping of transcript modules sharing high correlation coefficient (PCC > 0.7) with the identified metabolite modules in *Mallotus japonicus*.

Table S14. Gene ontology (GO) enrichment analysis of transcript modules sharing high correlation coefficient (PCC > 0.7) with the identified metabolite modules in *Mallotus japonicus*.

Table S15. KEGG pathway mapping of transcript modules TransM4 and TransM9 sharing high correlation coefficient (PCC > 0.7) with the metabolite module MetM7 containing rutin.

Table S16. List of transcripts, annotated as glucosyltransferases (GTs) and *O*-methyltransferases (OMTs), involved in the biosynthesis of bergenin in *Mallotus japonicus.* Transcripts from TransM5, TransM10, and TransM15 transcript modules, annotated as GTs and OMTs were selected.

Table S17. Accession number of the functionally characterized glucosyltransferases (GTs) and *O*-methyltransferases (OMTs) used for phylogenetic analysis.
