# Supplementary material for: Gene-Metabolite Network Analysis Revealed Tissue-Specific Accumulation of Therapeutic Metabolites in Mallotus japonicus
Source: Int J Mol Sci. 2021 Aug 17;22(16):8835. doi: 10.3390/ijms22168835 (PMC8396295; doi:10.3390/ijms22168835)
Supplement: Supplementary file 1 [file ijms-22-08835-s001.zip › Supplementary_Table S6 Summary of Trimmomatic output for Mallotus japonicus with legend.pdf]

Table S6 Summary of Trimmomatic output for *Mallotus japonicus*

	Young Leaf	Mature Leaf	Young Stem	Mature Stem	Bark	Central Cylinder	Inflorescence
**Input Read Pairs**	12,621,042	13,177,546	10,823,838	8,183,699	8,821,033	11,416,398	12,910,459
**Both Surviving**	11,502,563 (91.14%)	12,022,817 (91.24%)	9,849,490 (91.00%)	7,429,176 (90.78%)	8,047,221 (91.23%)	10,491,432 (91.90%)	11,795,640 (91.36%)
**Forward Only Surviving**	758.700 (6.01%)	778,256 (5.91%)	655,663 (6.06%)	515,876 (6.30%)	515,753 (5.85%)	620,133 (5.43%)	739,085 (5.72%)
**Reverse Only Surviving**	235,010 (1.86%)	244,716 (1.86%)	207,332 (1.92%)	155,539 (1.90%)	169,433 (1.92%)	201,643 (1.77%)	248,933 (1.93%)
**Dropped**	124,769 (0.99%)	131,757 (1.00%)	111,353 (1.03%)	83,108 (1.02%)	88,626 (1.00%)	103,190 (0.90%)	126,801 (0.98%)
