# Supplementary figures and images for: Gene-Metabolite Network Analysis Revealed Tissue-Specific Accumulation of Therapeutic Metabolites in Mallotus japonicus
Source: Int J Mol Sci. 2021 Aug 17;22(16):8835. doi: 10.3390/ijms22168835 (PMC8396295; doi:10.3390/ijms22168835)

## FLAVONOID BIOSYNTHESIS

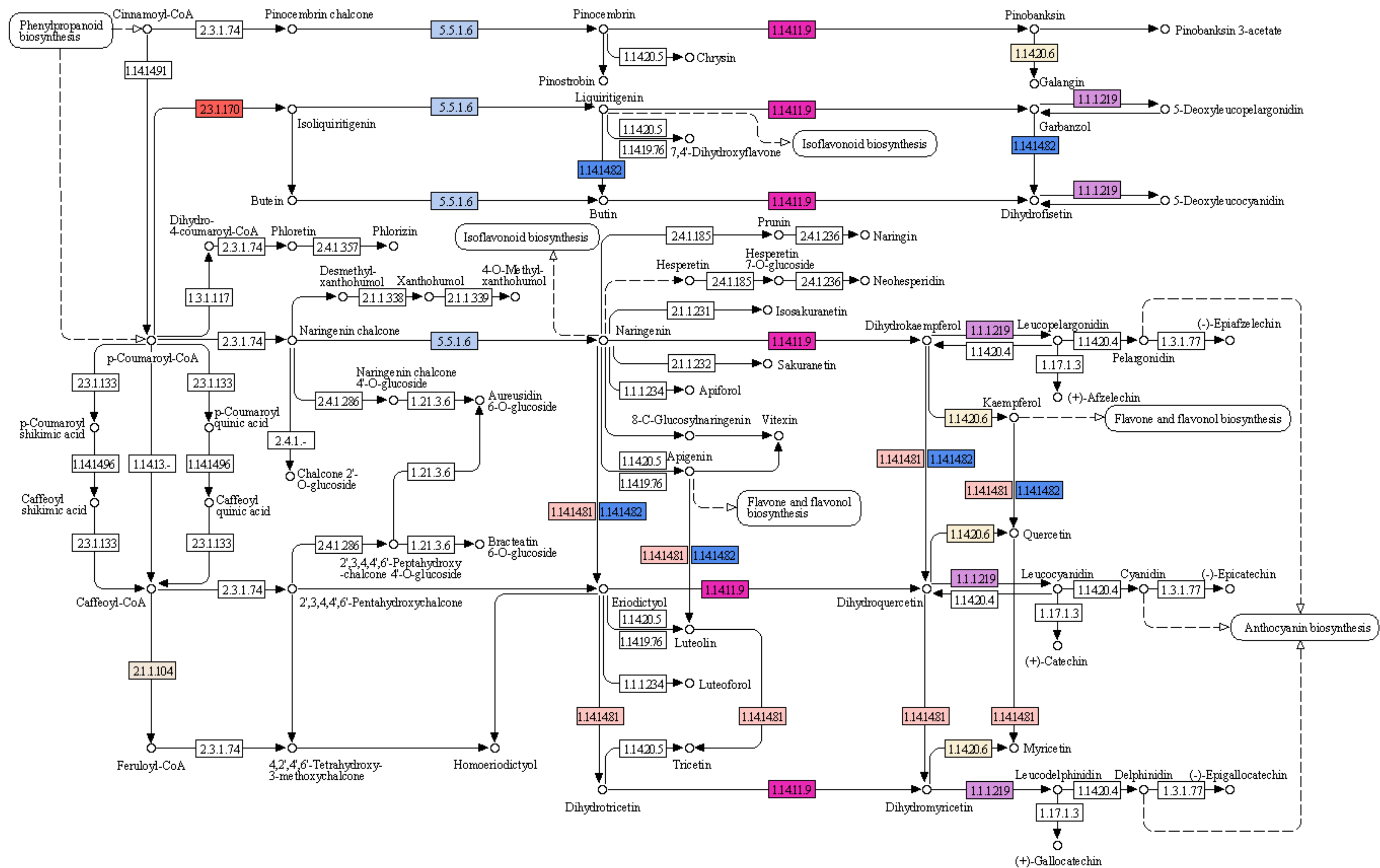

Supplement: Supplementary file 1 [file ijms-22-08835-s001.zip › Figure S7 Representation of flavonoid biosynthesis pathway TransM4 and TransM9 transcript modules with legend.pdf]
